# Exploring the mechanisms of tetrahydrocurcumin in ameliorating nonalcoholic steatohepatitis based on network pharmacology and gut microbiota analysis *in vivo* and *in vitro*

**DOI:** 10.3389/fmicb.2025.1576221

**Published:** 2025-06-17

**Authors:** Keyu Chen, Jianbo Wang, Shuang Luo, Yunyun Quan, Ping Wei, Jiali Fu, Jiali Ma, Yuying Yang, Yunten Liu, Zhichong Gao

**Affiliations:** ^1^Pharmacology of Chinese Medicine, Shaanxi University of Chinese Medicine, Xianyang, China; ^2^Translational Chinese Medicine Key Laboratory of Sichuan Province, Sichuan Institute for Translational Chinese Medicine, Sichuan Academy of Chinese Medicine Sciences, Chengdu, China; ^3^Key Laboratory of Pharmacodynamics and Material Basis of Chinese Medicine of Shaanxi Administration of Traditional Chinese Medicine, Xianyang, China; ^4^Engineering Research Center of Brain Health Industry of Chinese Medicine, Universities of Shaanxi Province, Xianyang, China; ^5^College of Food and Biological Engineering, Chengdu University, Chengdu, China; ^6^School of Pharmacy, Southwest Medical University, Luzhou, China

**Keywords:** nonalcoholic steatohepatitis, tetrahydrocurcumin, network pharmacology, 16S rRNA, intestinal flora imbalance

## Abstract

**Background:**

The prevalence of nonalcoholic steatohepatitis (NASH) is increasing every year, and there are very few approved therapeutic agents globally, making the search for potentially targeted therapeutic agents important.

**Aims:**

To investigate the anti-NASH effect of tetrahydrocurcumin (THC) and to further study the biological mechanism of THC anti-NASH from the perspective of intestinal flora.

**Methods:**

Seven-week-old female male C57BL/6J mice were randomly divided into two batches of six groups: (1) control group, (2) model group, (3) positive control group, (4) THC low-dose group, (5) THC medium-dose group, and (6) THC high-dose group. The first batch of mice were fed with high-fat chow for 16 weeks in the rest of the groups except the control group; and the second batch of mice were fed with MCS chow in the control group, and MCS chow in the rest of the groups. MCD feed for 4 weeks. Serum, feces and liver tissues were collected separately. In addition, NASH cell model was established by using free fatty acids to induce AML-12 cells. Network pharmacology, molecular docking, high-throughput sequencing, protein blotting, and real-time fluorescence quantitative PCR were used to investigate the mechanism of THC against NASH.

**Results:**

The intervention of THC improved the pathology of NASH, ameliorated liver injury, lowered lipid levels, and inhibited hepatic oxidative stress, inflammatory response and apoptosis compared with the high-fat feed-induced model group. In network pharmacology and animal experimental validation we found that THC reduced the expression of m RNA of PPARG, which may be the key to the improvement of NASH by THC. Intestinal flora analysis showed that THC altered the composition of the intestinal flora, which was characterized by a decrease in the proportion of Firmicutes/Bacteroidota.

**Conclusion:**

The results of this study suggest that THC exerts anti-NASH effects by improving lipid levels, decreasing oxidative stress, attenuating inflammatory responses, and increasing the anti-apoptotic capacity of liver cells, and its efficacy is importantly associated with decreasing the expression of PPARG and improving the intestinal flora. THC is expected to be a potential therapeutic agent for NASH.

## 1 Introduction

Non-alcoholic steatohepatitis (NASH) is the inflammatory subtype of non-alcoholic fatty liver disease (NAFLD), with approximately 25% of NAFLD patients progressing to NASH. Over time, this condition can advance to cirrhosis, end-stage liver disease, or require liver transplantation, significantly impacting patients’ physical and mental well-being (Sheka et al., 2020; Schwabe et al., 2020). While numerous drug trials for NAFLD/NASH are ongoing, approved treatments remain limited. Currently, behavioral interventions like weight loss, dietary control, and increased physical activity serve as the primary clinical strategies for managing NASH (Tokushige et al., 2021; Han et al., 2019). However, their long-term efficacy is hindered by hormonal and metabolic complexities. Given NASH’s intricate pathogenesis—where existing therapies typically target single pathways or mechanisms—a multi-target, multi-pathway approach may offer a novel direction for developing new potential treatments.

In recent years, the gut microbiota has emerged as a key focus of scientific inquiry. Given the specialized physiological connection between the intestines and the liver—the portal vein—metabolites produced by gut bacteria travel directly to the liver via this vascular pathway, influencing hepatic health. As a result, the relationship between the gut microbiota and liver conditions has drawn increasing attention, with growing evidence highlighting its potential role as a critical factor in NASH pathogenesis. This perspective offers new insights into understanding NASH development (Gomaa, 2020; Kim et al., 2020). Research has linked gut microbiota dysbiosis to insulin resistance, inflammation, obesity, and metabolic dysfunction—hallmarks of NASH (Pushpanathan et al., 2019). In animal studies, alterations in the gut microbiota have been shown to impact liver inflammation and steatosis in NASH models, processes tightly tied to metabolism-related biomarkers (Zheng et al., 2019; Yang et al., 2021; Sun et al., 2021). Thus, targeting the gut microbiota represents a promising therapeutic strategy for NASH.

**Figure 1:**
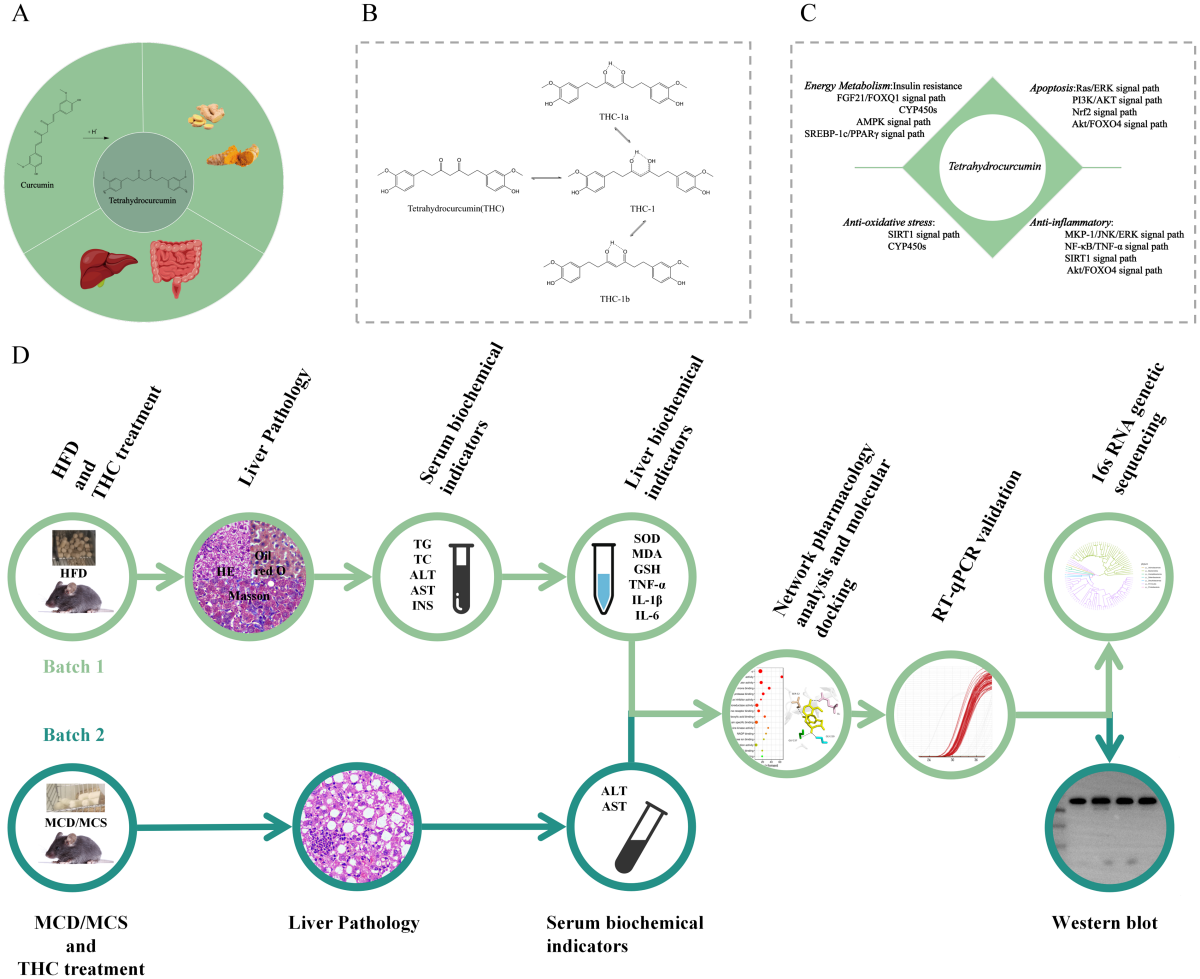
Introduction and graphical summary of THC. **(A)** Source pathway of THC. **(B)** Structure of THC. **(C)** Pathways related to the pharmacological activity of THC. **(D)** Experimental procedure.

THC, a major bioactive metabolite of the natural antioxidant curcumin (Figure 1A), owes its potent antioxidant and anti-inflammatory properties to the β-diketone moiety in its structure. This moiety breaks the C–C bond at the reactive methylidene carbon between two carbonyl groups (Figure 1B), enabling pharmacological activities such as modulating oxidative stress, inflammation, cell proliferation, apoptosis, and immunity (Figure 1C). Preclinical studies have demonstrated its protective effects against inflammation, obesity-related insulin resistance, hepatic steatosis, and diabetes (Li et al., 2019; Lai et al., 2020; Kim et al., 2009; Karthikesan et al., 2010). Further research shows that THC reduces hepatic lipid accumulation, mitigates high-fat diet–induced oxidative stress in the liver, alleviates liver injury, and modulates the intestinal microbiota (Gao et al., 2022; Jarukamjorn et al., 2019; Yuan et al., 2020). Notably, no prior studies have investigated whether THC improves NASH by regulating the intestinal flora. Therefore, this study aimed to: (1) assess the pharmacodynamic effects of THC in a NASH mouse model; (2) explore its potential mechanisms via network pharmacology and molecular docking; and (3) analyze intestinal flora diversity through high-throughput sequencing. These approaches collectively provide a robust foundation for elucidating how THC ameliorates NASH via the intestinal microbiota (Figure 1D).

## 2 Materials and methods

### 2.1 Animal treatment and sample collection

Seven-week-old C57BL/6J mice (16–24 g body mass) were purchased from Guangdong Viton Lihua Laboratory Animal Technology Co., Ltd. (Guangdong, China; License No. SCXK-2022-0063). Mice were housed in an SPF-grade barrier facility at the Experimental Animal Center of Sichuan Academy of Traditional Chinese Medicine (Sichuan, China; License No. SYXK-2023-0100), maintained at 22 ± 2°C, 50 ± 5% humidity, and a 12-h light/dark cycle, with ad libitum access to food and water. All experimental procedures were approved by the Laboratory Animal Ethics Committee of Sichuan Academy of Traditional Chinese Medicine (Ethics Approval No. DWSYLL-2023-031).

Following a 1-week acclimation period, all mice were randomly assigned to 2 batches comprising 6 groups: control (Con), model (Mod), positive control (Mod + Silymarin), low-dose THC (Mod + THC50), medium-dose THC (Mod + THC100), and high-dose THC (Mod + THC200). Batch 1: 8 mice/group (50% male, 50% female; housed in sex-separated cages). The Con group received standard mouse chow (containing corn, wheat, fish meal, chicken meal, soybean oil, sunflower oil, amino acids, vitamins, minerals, etc.), while all other groups were fed a high-fat diet (HFD, D12492; 20% protein, 20% carbohydrate, 60% fat) for 16 weeks. Mouse body weights were recorded weekly, and fasting blood glucose levels were measured with a glucometer in the 16th week. Batch 2: 12 male mice/group. The Con group received methionine-choline-sufficient (MCS) chow, whereas other groups were fed methionine-choline-deficient (MCD) chow for 4 weeks. All diets (HFD, MCD, and MCS) were purchased from Xiaoshu Youtai (Beijing) Biotechnology Co., Ltd. The Con and Mod groups received 0.5% CMC-Na via gavage; low-, medium-, and high-dose THC groups were administered THC suspensions at 50, 100, and 200 mg/kg, respectively; the positive control group received silymarin suspension (200 mg/kg). THC suspension preparation: weighs 0.50 g, 0.25 g, and 0.125 g of THC into a mortar and pestle. Add 25 mL of 0.5% carboxymethylcellulose sodium (CMC-Na) solution, then stir continuously to form a homogeneous suspension. Store at 4°C and shake well before use. Silymarin suspension preparation: weighs 0.50 g of silymarin into a mortar, add 25 mL of 0.5% CMC-Na solution, and stir continuously to prepare the suspension. Store at 4°C and shake vigorously prior to use. 0.5% CMC-Na solution preparation: weighs 0.50 g of sodium carboxymethylcellulose powder and slowly add it to a beaker containing 100 mL of ultrapure water while stirring continuously. Allow the mixture to stand at 4°C overnight for complete dissolution, then filter and dispense into sterile containers for use. THC (Item No. 20191201, mass fraction ≥99%) was provided by the Institute of Pharmacy, Sichuan Academy of Traditional Chinese Medicine. Silymarin (Approval No. HJ201811067) was purchased from MADAUS GMBH, Germany. CMC-Na (Item Number R008134; Shanghai Yi En Chemical Technology Co., Ltd) was purchased for use in this study.

**Figure 2:**
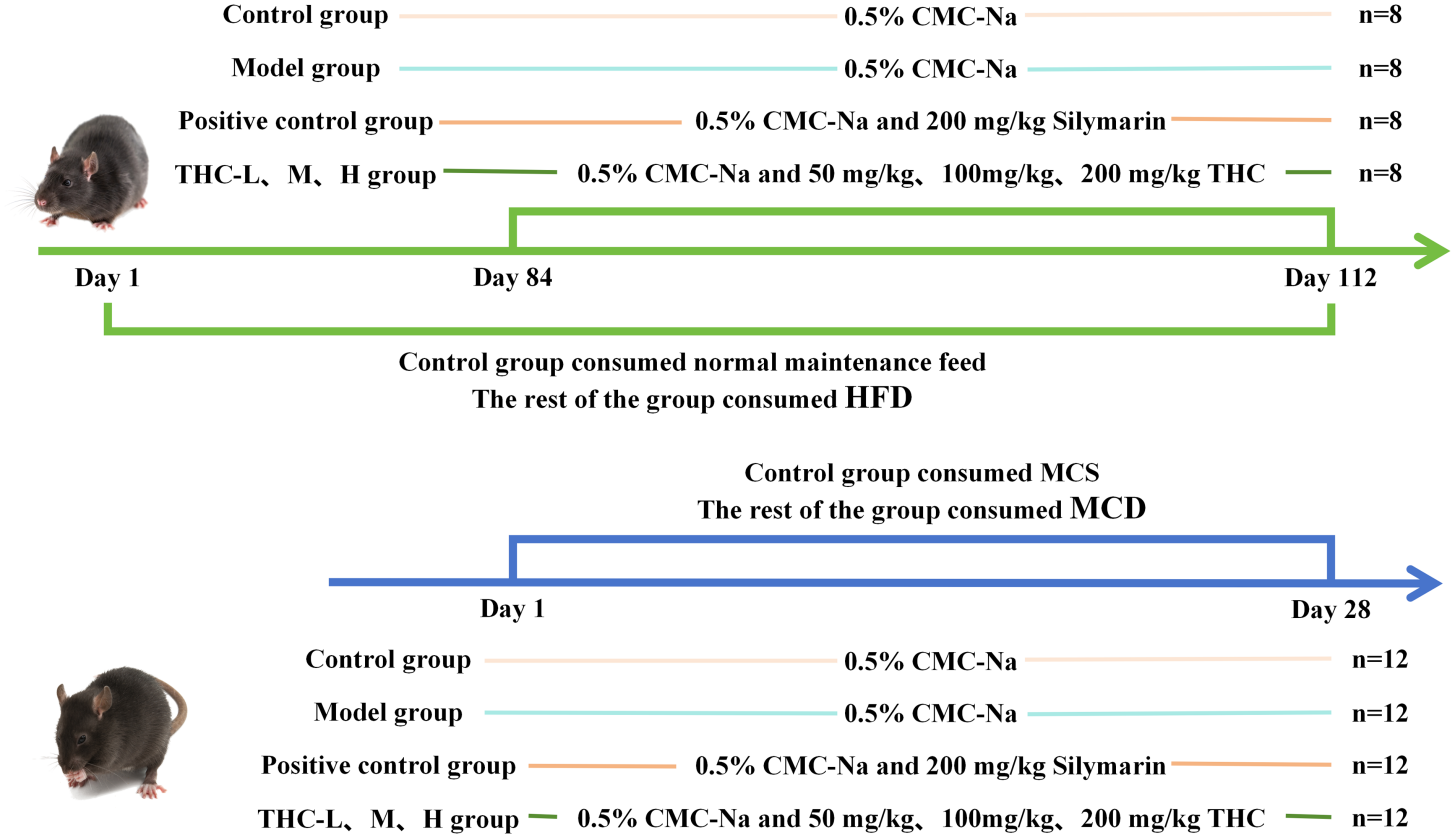
Animal modeling and therapeutic procedures.

After 4 weeks of treatment, all mice were fasted for 12 h. Blood was collected via retro-orbital puncture, centrifuged at 3,000 rpm for 10 min at 4°C, and serum was stored at −20°C for later analysis. Mice were euthanized by cervical dislocation, and livers were dissected, rinsed with ice-cold saline, and blotted dry with filter paper to remove excess moisture. Liver morphology was visually assessed, wet weights were recorded, and the liver index was calculated as (liver wet weight [g]/body weight [g]) × 100%. Following weighing, each liver was divided into two portions: one fixed in 4% paraformaldehyde for histology, and the remaining tissue frozen at −80°C for subsequent analysis (Figure 2).

### 2.2 Histopathological examination

Following gradient dehydration, fixed liver tissues were embedded and sectioned. Pathological sections were stained with hematoxylin and eosin (HE) and Masson trichrome for morphological analysis. Images of 400× magnification were captured using a BA210Digital trinocular video microscopy system (McAudi, China). Frozen liver tissues were trimmed, embedded, and sectioned, then post-fixed in formaldehyde for 10 min before staining with Oil Red O solution (Bomei, Hefei, China). Hepatic lesions in each group were evaluated using the NAFLD Activity Score (NAS), with criteria detailed in Table 1.

**Table 1:** NAS scoring criteria.

Score	Pathological appearance		
	Steatosis	Lobule inflammation	Hepatocyte ballooning
0	<5%	No foci	None
1	5–33%	<2 foci per 200× field	Few balloon cells
2	33–66%	2–4 foci per 200× field	Many
3	>66%	>4 foci per 200× field	—

### 2.3 Cell processing

**Figure 3:**
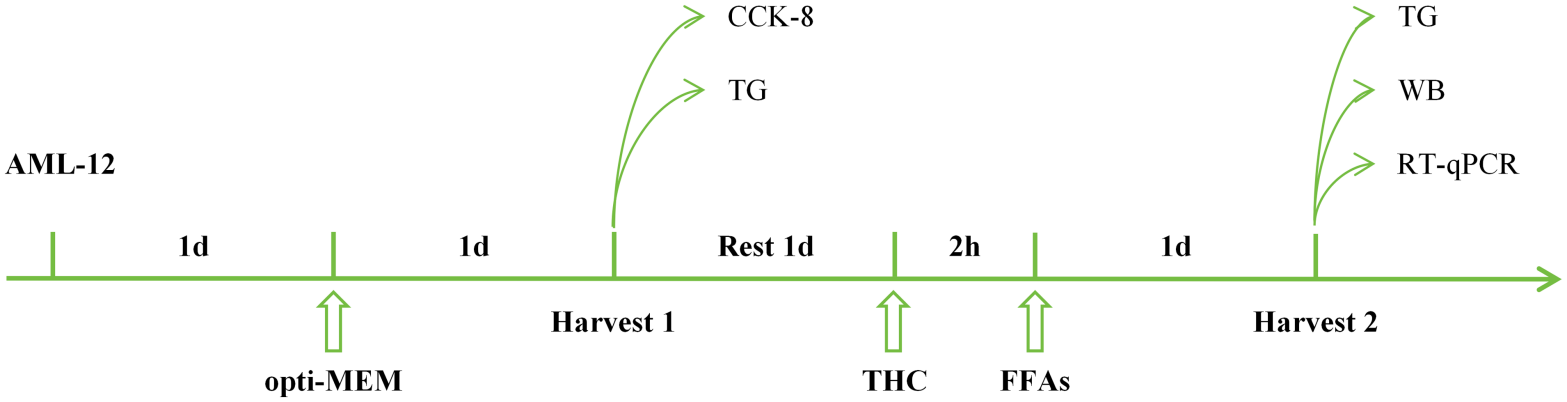
Cellular experimental procedures.

The mouse normal liver cell line AML-12 was purchased from the cell bank of Shanghai Fuhang Biotechnology Co. After successful resuscitation, cells were induced with opti-MEM reduced-serum medium for 24 h, then switched to complete medium for normal culture to activate liver function. A 6 mM free fatty acid (FFA) solution was prepared as a 2:1 ratio of sodium oleate to sodium palmitate. AML-12 cells were seeded in 6-well plates at 5 × 10⁵ cells/well, with groups including control (Con), model (Mod), THC low (THC-L, 1.0 μM) and high (THC-H, 2.0 μM) dose groups, and THC high dose + AMPK inhibitor (THC-C, 10 μM), with 3 wells per group. After overnight stabilization, the old medium was removed, and fresh DMEM/F12 was added to each well. FFAs were diluted to 1 mM in serum-free DMEM/F12, while THC and the AMPK inhibitor were dissolved in DMSO and diluted in serum-free DMEM/F12. THC-L and THC-H groups received corresponding THC doses; all other groups received an equal volume of vehicle. After 1.5 h, the AMPK inhibitor was added to the THC-C group, with other groups receiving vehicle. After 0.5 h, the Con group was treated with serum-free medium, while non-Con groups were exposed to 1 mM FFAs for 24 h in an incubator. Following incubation, medium was discarded, cells were washed with PBS, harvested by scraping, centrifuged to retain pellets, resuspended in 300 μL PBS, and stored at −80°C (Figure 3).

### 2.4 Biochemical index

Serum levels of alanine transaminase (ALT), aspartate transaminase (AST), triglycerides (TG), total cholesterol (TC), low-density lipoprotein cholesterol (LDL-C), and insulin (INS) were measured using ELISA kits from Jiangsu Enzyme Immuno (Jiangsu, China). Frozen tissue was accurately weighed, diced, and homogenized in 9 volumes of ice-cold saline using an ice-water bath to prepare a 10% tissue homogenate. The homogenate was centrifuged at 4°C for 10 min at 10,000 rpm, and the supernatant was immediately stored on ice for subsequent analysis. Liver tissue levels of superoxide dismutase (SOD), malondialdehyde (MDA), and glutathione (GSH) were assayed with kits from Nanjing Jianjian (Nanjing, China) and Wuhan Eliot (Wuhan, China). Mouse livers were thawed and centrifuged at 4°C, 15,000 rpm for 5 min to collect the supernatant. Proinflammatory cytokine levels (TNF-α, IL-1β, and IL-6) in liver tissues were quantified using kits from Chengdu Novo (Chengdu, China).

### 2.5 RT-qPCR

Liver tissues were homogenized using a tissue homogenizer, and total RNA was extracted from the homogenates. RNA quality and quantity were assessed with an ultra-micro spectrophotometer (Hangzhou Ausheng, Zhejiang, China). Weighs 10–20 mg of tissue and add 500 μL of Buffer RL1 (Hangzhou Ausheng, Zhejiang, China). Homogenize the sample with a tissue homogenizer, then transfer the homogenate to a DNA-Cleaning Column. Centrifuge at 12,000 rpm for 2 min and collect the supernatant. Add 1.6 volumes of Buffer RL2 to the supernatant and mix gently. Transfer the mixture to an RNA-only column, centrifuge, and discard the flow-through. Replace the purification column into the collection tube, load all remaining mixture onto the column, and centrifuge again to discard waste. Wash the column by adding 500 μL of buffer RW1, centrifuging, and removing the waste. Next, add 700 μL of buffer RW2, centrifuge, discard the waste, and repeat this wash step once. Finally, add 50–200 μL of 65°C preheated RNase-free ddH_2_O to the center of the column membrane. Incubate at room temperature for 2 min, then centrifuge to elute and collect the RNA solution. Following extraction, total RNA was reverse-transcribed into cDNA using a first-strand cDNA synthesis kit (Chengdu Rongwei, Sichuan, China) according to the manufacturer’s instructions. PCR amplification was performed using gene-specific primers (Table 2), with GAPDH serving as the housekeeping gene. Relative gene expression levels were calculated using the 2^−ΔΔCT^ method.

**Table 2:** Primer sequence.

Gene	Forward primer	Reverse primer	Product length	Gene ID
BCL2	GCTACCGTCGTGACTTCGC	CCCCACCGAACTCAAAGAAGG	147	12,043
Bax	TGAAGACAGGGGCCTTTTTG	AATTCGCCGGAGACACTCG	140	12,028
PPARG	GGAAGACCACTCGCATTCCTT	GTAATCAGCAACCATTGGGTCA	121	19,016
CYP7A1	GCTGTGGTAGTGAGCTGTTG	GTTGTCCAAAGGAGGTTCACC	78	13,122
CYP8B1	CCTCTGGACAAGGGTTTTGTG	GCACCGTGAAGACATCCCC	112	13,124
FXR	GCTTGATGTGCTACAAAAGCTG	CGTGGTGATGGTTGAATGTCC	110	20,186
ACC	ATGGGCGGAATGGTCTCTTTC	TGGGGACCTTGTCTTCATCAT	148	107,476
AMPK	TTCGGGAAAGTGAAGGTGGG	TCTTCTGCCGGTTGAGTATCT	76	105,787
mTOR	ACCGGCACACATTTGAAGAAG	CTCGTTGAGGATCAGCAAGG	110	56,717
GAPDH	CAGTGGCAAAGTGGAGATTGTTG	TCGCTCCTGGAAGATGGTGAT	169	14,433

### 2.6 Western blot

Cells were lysed by adding 100 μL of RIPA buffer containing PMSF (1:100 ratio), scraped from plates with a cell scraper, and kept on ice. Cell lysates were quantified using a BSA protein assay kit. For sample preparation, lysates were mixed with SDS loading buffer in new EP tubes, boiled at 95–100°C for 10 min, cooled, and centrifuged for 5 min; 20–40 μg of total protein was loaded per well. Gels were prepared with 10% or 12% separating gel and 5% stacking gel. After electrophoretic separation, proteins were transferred to a PVDF membrane, which was then blocked. The membrane was incubated with primary antibody diluted in 5% skim milk-TBST at 37°C for 2 h, washed, and subsequently incubated with secondary antibody in the same diluent at 37°C for 1 h. Following final washes, protein bands were visualized using a gel imaging system. Relative protein expression was calculated as the ratio of target protein to housekeeping protein signals.

### 2.7 Network pharmacology analysis

To identify potential targets of THC, we first predicted targets using databases including PharmMapper, BATMAN, and SwissTargetPrediction. Next, we searched for NASH-associated disease targets by inputting “nonalcoholic steatohepatitis” as a keyword into databases such as NCBI, GeneCards, OMIM, DisGeNET, DrugBank, and TTD. Third, intersecting targets between THC and NASH were uploaded to the STRING 11.5 platform for protein–protein interaction (PPI) network analysis. Fourth, these shared targets underwent functional enrichment analysis—including GO molecular function (MF), biological processes (BP), cellular components (CC), and KEGG pathways—via the Metascape database. Finally, CytoScape 3.9.1 software was used to construct PPI networks for monomer-disease relationships, core target screening, and monomer-target-pathway interactions. For molecular docking, THC was defined as the ligand in AutoDockTools 1.5.7. Key target structures (PDB IDs) were retrieved from the UniProt database, processed in PyMOL 2.6 (e.g., removing heteroatoms), and receptor boxes were configured in AutoDockTools 1.5.7 with recorded parameters. Docking was performed using AutoDock Vina 1.2.3, and results were visualized in PyMOL 2.6 to analyze ligand-receptor interactions.

### 2.8 16S rRNA sequencing analysis

Fecal genomic DNA was extracted using a DNA isolation kit, and its concentration and purity were assessed via NanoDrop 2000 and agarose gel electrophoresis. The V3-V4 region of the 16S rRNA gene was amplified using universal primers 343F and 798R for diversity analysis via PCR. Library preparation, sequencing, and initial data analysis were conducted by Shanghai Ouyi Biomedical Technology Co., Ltd. Quality control steps—including filtering, denoising, sequence assembly, and chimeric sequence removal—were performed using default parameters in QIIME 2 (2020.11), yielding representative sequences and ASV (Amplicon Sequence Variant) abundance tables. Representative sequences for each ASV were selected within the QIIME 2 pipeline and annotated against the Silva (version 138) database for taxonomic classification.

### 2.9 Statistical analysis

Experimental data were analyzed using SPSS 25 software, with results reported as mean ± standard deviation. Data from each group were first tested for normality. Normally distributed data underwent one-way analysis of variance (ANOVA). For homogeneous variance, post-hoc comparisons used the least significant difference (LSD) test, and independent samples *t*-tests compared two groups; non-parametric rank sum tests were applied for non-homogeneous variance. Statistical significance was defined as *p* < 0.05.

## 3 Results

### 3.1 THC attenuates liver injury and lipid elevation in NASH mice

**Figure 4:**
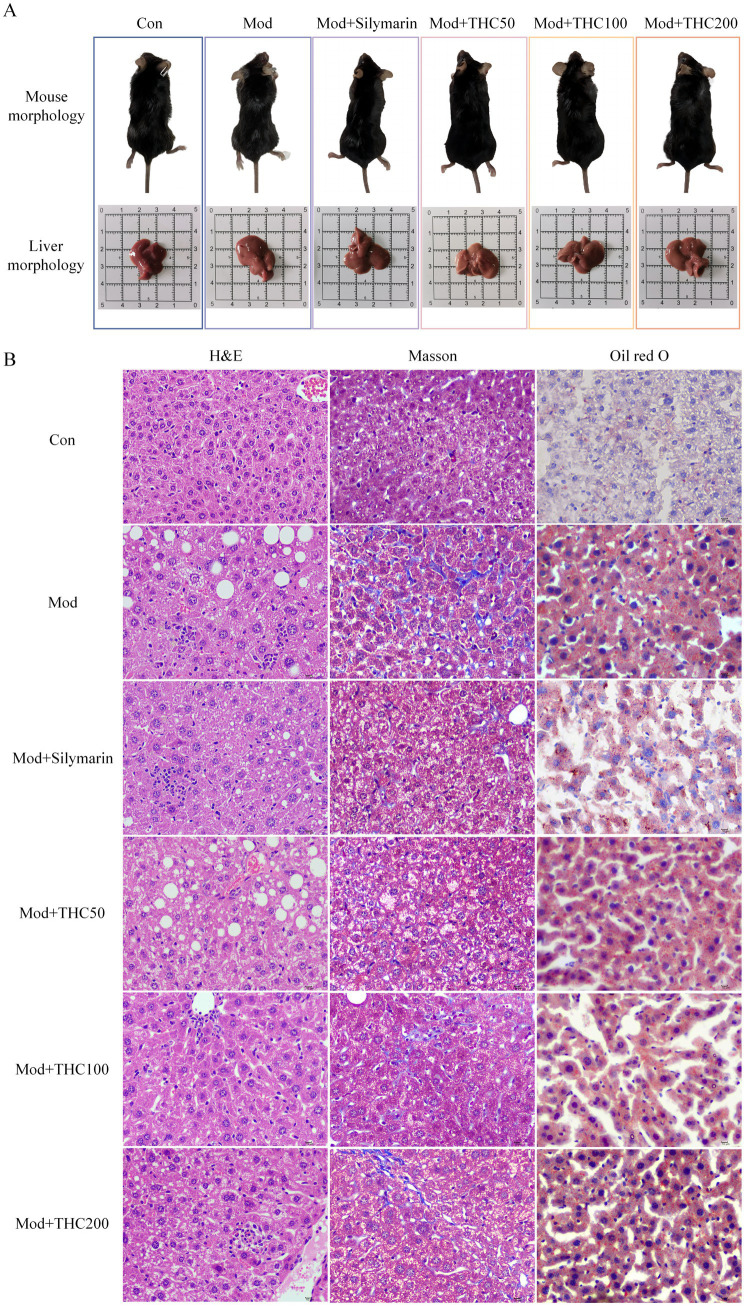
Effect of THC on body morphology, liver morphology and extent of lesions in NASH mice. **(A)** Typical images of body and liver morphology in mice. **(B)** Typical images of HE staining, Masson staining and Oil Red O staining of liver tissue (magnification, 400×).

**Figure 5:**
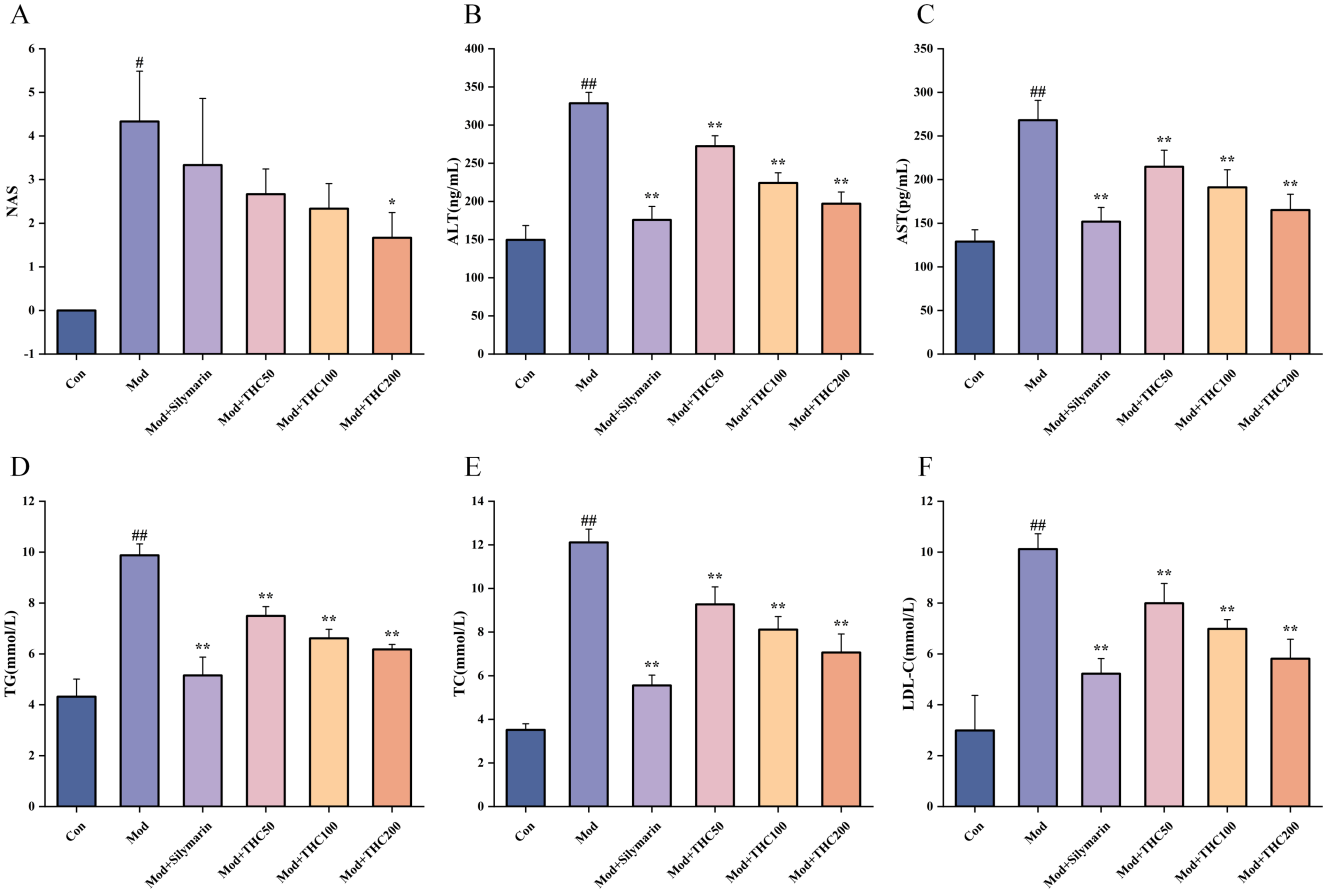
Effects of THC on NAS score, liver function and lipids in NASH mice. **(A)** NAS score on HE-stained liver sections (*n* = 3). **(B)** Effect of THC on serum ALT. **(C)** Effect of THC on serum AST. **(D)** Serum TG level. **(E)** Serum TC level. **(F)** Serum LDL-C level. Compared with Con group, ^#^*p* < 0.05 and ^##^*p* < 0.01; compared with Mod group, ^*^p < 0.05 and ^**^p < 0.01.

When compared to the control group, the model group exhibited significantly lower body weights and higher liver indices (Table 3). THC intervention increased mouse body weight and reduced liver indices relative to the model group, with representative morphological images shown in Figure 4A. HE and Masson staining (Figure 4B) revealed that model group livers displayed vacuolar degeneration, severe steatosis, extensive punctate necrosis with inflammatory infiltration, increased collagen fiber deposition, expanded fibrosis areas, and a NAS score >4 (Figure 5A)—hallmarks of successful NASH modeling. These pathological changes and NAS scores were dose-dependently ameliorated by THC. Serum ALT and AST analyses (Figures 5B,C) showed significantly elevated levels in the model group vs. control, with THC treatment reducing both enzymes in a dose-dependent manner, indicating improved hepatic injury. Oil Red O staining (Figure 4B) demonstrated increased hepatic lipid accumulation in the model group, which was attenuated by THC and the positive control. Serum lipid profiles (TG, TC, LDL-C; Figures 5D–F) mirrored this pattern: model group levels were significantly higher than control, while the positive control and THC groups showed significant reductions, consistent with the ALT/AST trends and suggesting lipid-regulatory effects of THC.

**Table 3:** Effect of THC on body weight and liver index in NASH mice.

Group	Dosages (mg/kg)	Body weight ( $\bar{X}\pm \mathrm{SD}$ )	L/W index ( $\bar{X}\pm \mathrm{SD}$ )
Con	—	20.67 ± 1.45	4.20 ± 0.16
Mod	—	19.76 ± 2.11	5.52 ± 0.51^##^
Mod + Silymarin	200	21.84 ± 2.19	5.29 ± 0.32
Mod + THC50	50	22.09 ± 2.83^*^	5.00 ± 0.38^*^
Mod + THC100	100	22.65 ± 1.72^**^	4.81 ± 0.44^**^
Mod + THC200	200	22.84 ± 2.11^**^	4.74 ± 0.19^*^

Compared with Con group, ^#^*p* < 0.05 and ^##^*p* < 0.01; compared with Mod group, ^*^p < 0.05 and ^**^p < 0.01.

**Figure 6:**
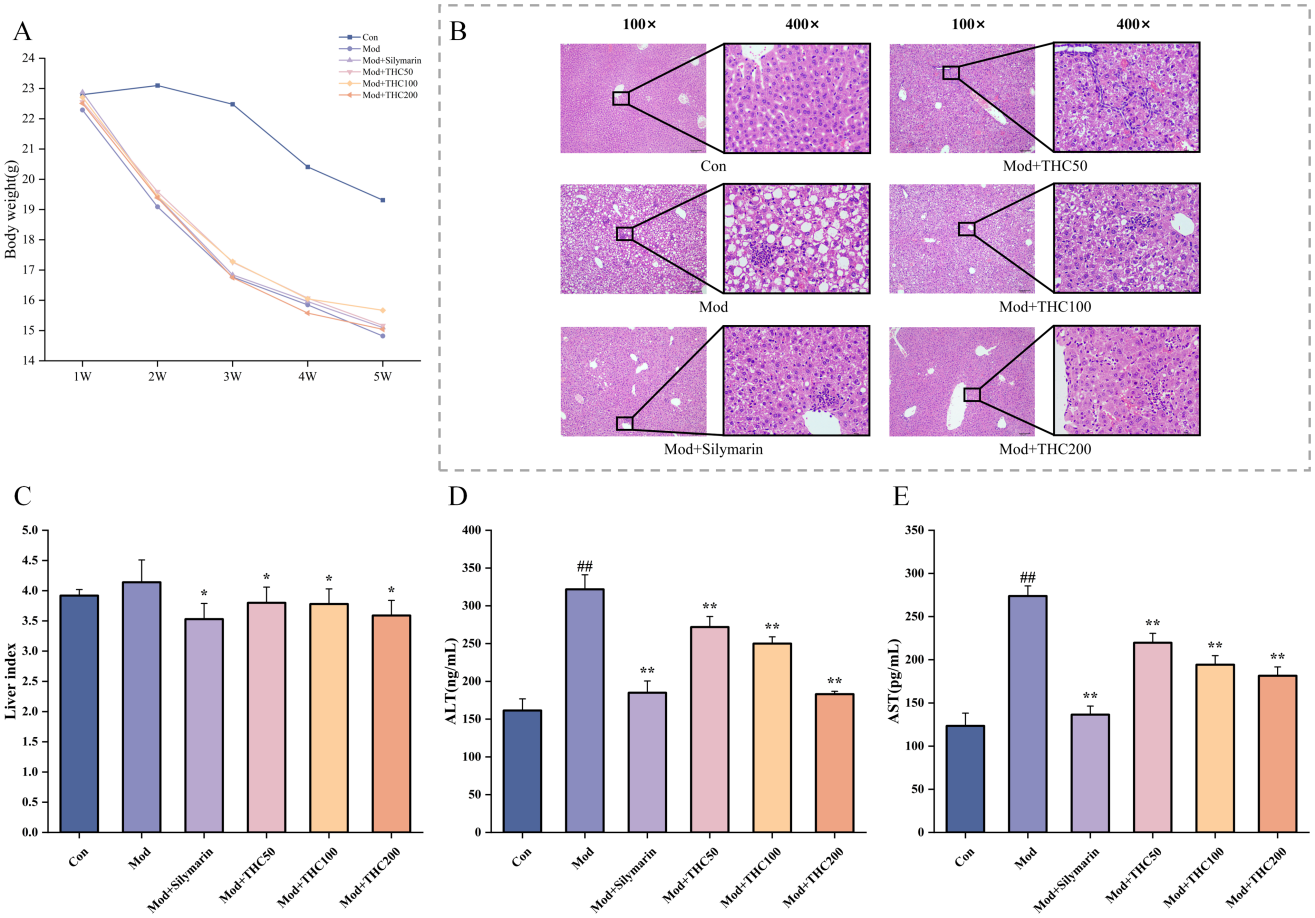
Effects of THC on body weight, liver pathology, NAS score and liver function in MCD-fed mice. **(A)** THC delayed the rate of body weight loss in MCD mice. **(B)** Typical images of HE staining of liver tissue in MCD-fed mice. **(C)** NAS scoring of HE-stained liver sections by THC (*n* = 6). **(D)** Effect of THC on serum ALT. **(E)** Effect of THC on serum AST. Compared with Con group, ^#^*p* < 0.05 and ^##^*p* < 0.01; compared with Mod group, ^*^p < 0.05 and ^**^p < 0.01.

Mice fed a methionine-choline-deficient (MCD) diet exhibited progressive weight loss, though THC intervention mitigated this decline, reduced liver lesion severity, and lowered liver indices (Figures 6A–C). Additionally, THC treatment dose-dependently decreased serum alanine transaminase (ALT) and aspartate transaminase (AST) levels in MCD mice (*p* < 0.01; Figures 6D,E), indicating improved liver injury resolution.

### 3.2 THC ameliorates insulin resistance, oxidative stress, inflammatory response, and apoptosis in NASH mice

**Figure 7:**
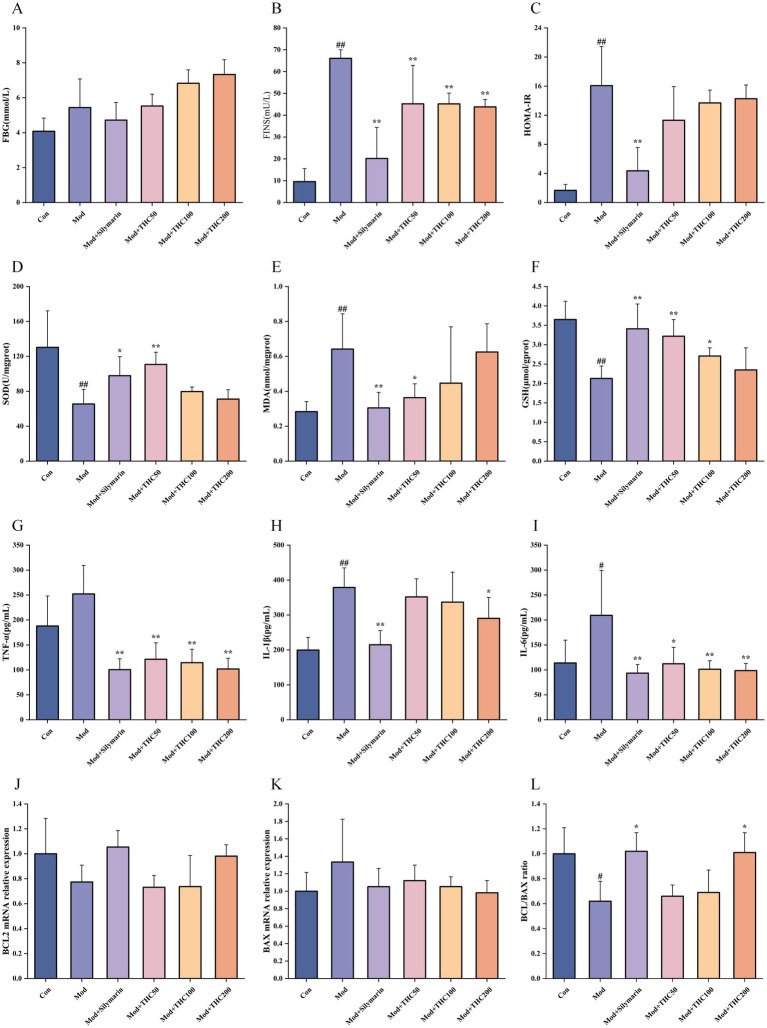
Effect of THC on biochemical indices in NASH mice. **(A)** Fasting blood glucose values. **(B)** Fasting insulin levels. **(C)** Insulin resistance index of mice in each group. **(D)** Liver SOD activity. **(E)** Liver MDA content. **(F)** Liver GSH content. **(G)** Liver TNF-α content. **(H)** Hepatic IL-1β content. **(I)** Hepatic IL-6 content. **(J)** mRNA expression of BCL-2 in liver. **(K)** mRNA expression of BAX in liver. **(L)** Ratio of BCL-2 to BAX. Compared with Con group, ^#^*p* < 0.05 and ^##^*p* < 0.01; compared with Mod group, ^*^p < 0.05 and ^**^p < 0.01.

Fasting blood glucose was measured using a glucometer, and the homeostasis model assessment of insulin resistance (HOMA-IR) was calculated using fasting serum insulin levels (Figures 7A–C). THC significantly reduced serum insulin and trended toward lowering HOMA-IR in model mice. Liver tissue analyses (Figures 7D–F) revealed that compared to controls, model mice had decreased superoxide dismutase (SOD) activity and glutathione (GSH) levels, alongside increased malondialdehyde (MDA)—markers of oxidative stress. THC treatment reversed these changes, increasing SOD/GSH and decreasing MDA in a dose-dependent manner. Proinflammatory cytokine levels (Figures 7G–I) were elevated in model livers vs. controls, with TNF-α, IL-1β, and IL-6 significantly reduced by 200 mg/kg THC. Regarding apoptosis-related gene expression (Figures 7J–L), a high-fat diet downregulated BCL-2 mRNA and upregulated BAX mRNA, decreasing the BCL-2/BAX ratio—indicative of hepatic cell apoptosis. High-dose THC reversed this imbalance, restoring the BCL-2/BAX ratio to near-control levels.

### 3.3 Potential core targets of THC against NASH

**Figure 8:**
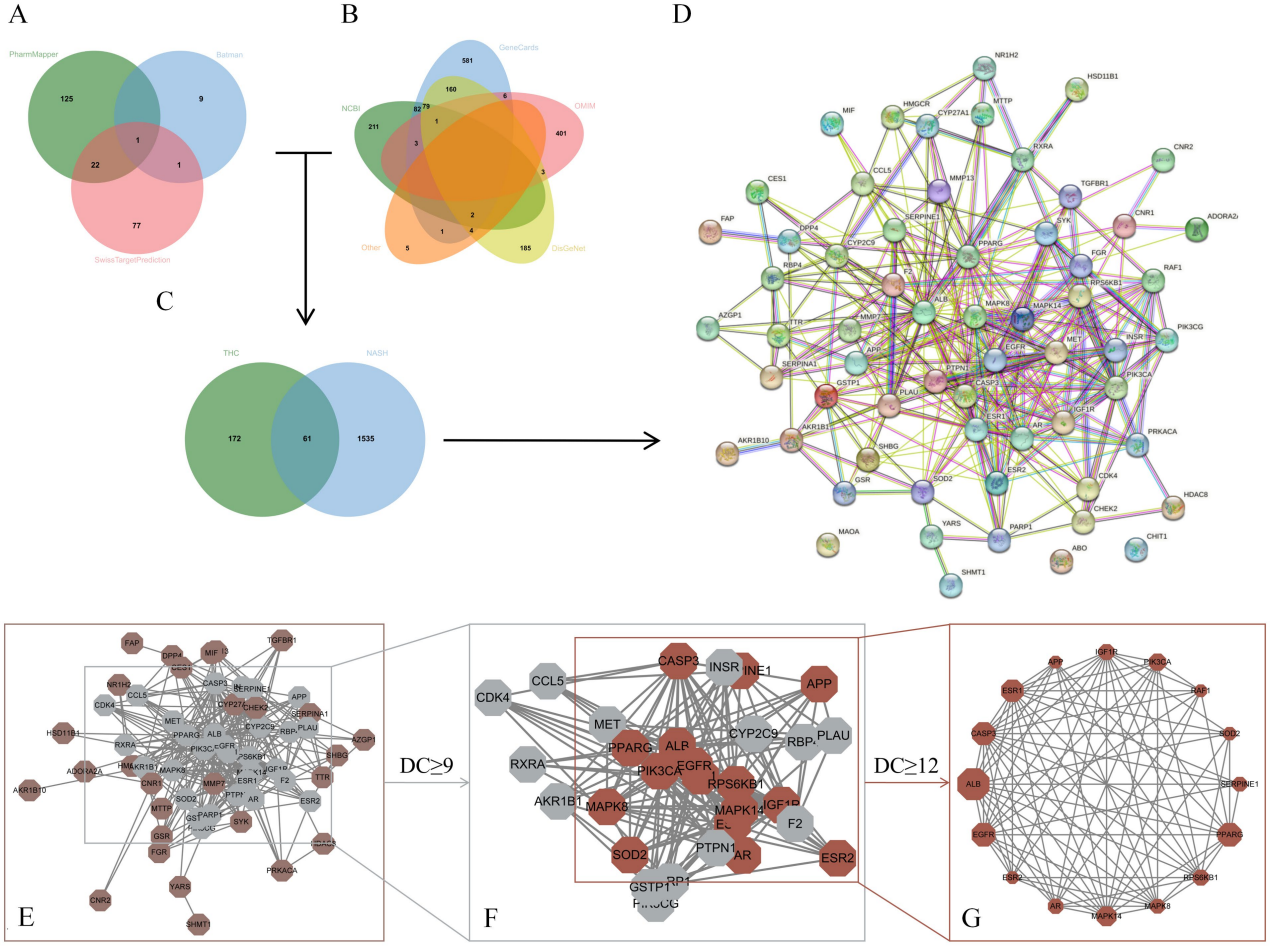
Core target screening process for THC anti-NASH. **(A)** Potential targets of THC. **(B)** NASH-related targets. **(C)** Wayne diagram of intersecting targets. **(D)** Protein interaction network diagram. **(E)** PPI network diagram visualization. **(F)** Initial screening of core targets. **(G)** Core target network diagram.

A total of 233 targets were obtained by integrating the target information predicted by three databases, including PharmMapper database, BATMAN database, and Swiss TargetPrediction, after eliminating duplicate values (Figure 8A). By aggregating data from NCBI, GeneCards, OMIM, DisGeNET, DrugBank, and TTD, we identified 1,596 NASH-associated targets (Figure 8B). Through subsequent screening, 61 intersecting targets between THC and NASH were obtained (Figure 8C). These 61 targets were uploaded to STRING 11.5 with a medium confidence threshold of 0.400, generating a protein–protein interaction (PPI) network with 61 nodes and 304 edges (Figure 8D). The PPI enrichment *p*-value was 1.0 × 10^−16^. The PPI network TSV file was downloaded and imported into Cytoscape 3.9.1 for visualization and core target identification (Figures 8E,G). Network E, composed of 58 nodes and 304 edges, was analyzed using the cytoNCA plugin to calculate degree centrality (DC). Networks F and G were filtered based on DC ≥9 and DC ≥12, respectively. Network G, with 16 nodes and 86 edges, was sorted by DC in descending order. The 16 resulting targets (ALB, PPARG, EGFR, CASP3, ESR1, MAPK14, PIK3CA, IGF1R, RPS6KB1, AR, MAPK8, SERPINE1, SOD2, RAF1, ESR2, and APP) are potential core targets.

### 3.4 GO enrichment analysis and KEGG pathway enrichment

**Figure 9:**
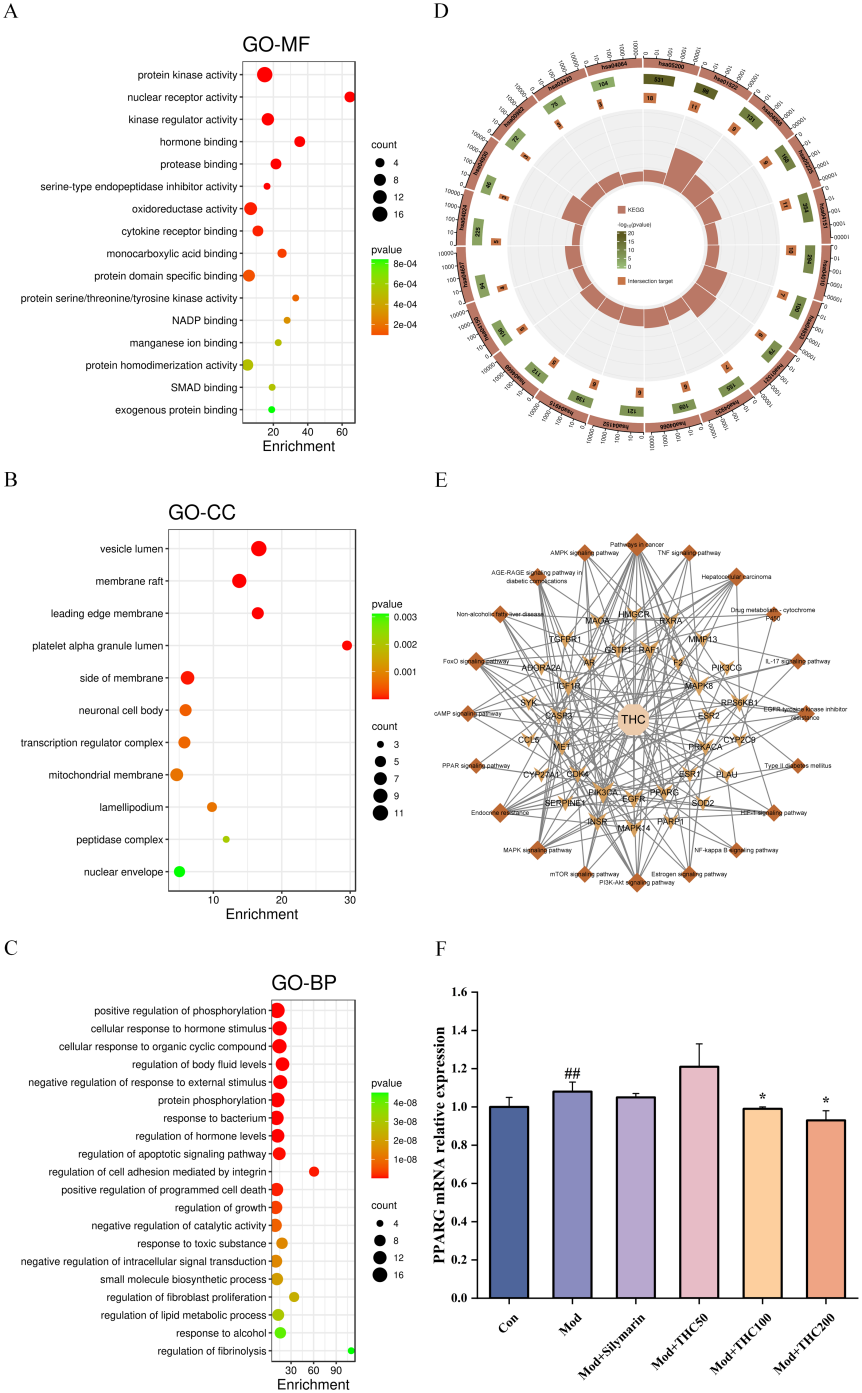
GO functional enrichment analysis and KEGG pathway enrichment analysis. **(A)** GO-MF. **(B)** GO-BP. **(C)** GO-CC. **(D)** KEGG. **(E)** Monomer-target-pathway network diagram. **(F)** Relative PPARG mRNA expression in the liver of mice in each group. Compared with Con group, ^##^*p* < 0.01; compared with Mod group, ^*^p < 0.05.

The 61 intersecting targets were imported into the Metascape database for GO molecular function (GO-MF), GO biological process (GO-BP), GO cellular component (GO-CC), and KEGG pathway analyses, with a *p*-value threshold of 0.01 and a minimum enrichment of 1.5. This analysis yielded 71, 646, 48, and 143 items for GO-MF, GO-BP, GO-CC, and KEGG, respectively. After exporting the data, the top 20 items were sorted by ascending *p*-value and used to generate GO enrichment bubble diagrams and KEGG enrichment circle diagrams using a microbiology website and Inkscape 1.2 software (Figures 9A–D). GO-MF analysis suggested that THC-related targets may improve NASH through molecular functions such as nuclear receptor activity, hormone binding, oxidoreductase activity, cytokine receptor binding, NADP binding, and SMAD binding. GO-BP analysis indicated that biological processes associated with THC-related targets for NASH improvement included positive regulation of phosphorylation, cellular response to hormonal stimuli, regulation of hormone levels, regulation of the apoptosis signaling pathway, and regulation of lipid metabolic processes (Figures 9A,C). KEGG enrichment results showed that THC may exert its effect on NASH improvement through signaling pathways such as the PI3K-Akt, MAPK, AGE-RAGE, AMPK, and TNF signaling pathways (Figure 9D). The top 20 signaling pathways ranked by ascending *p*-value are presented in Table 4.

**Table 4:** KEGG pathway analysis results.

Term ID	Path name	*p*-value
hsa05200	Pathways in cancer	1.27845 × 10^−17^
hsa01522	Endocrine resistance	8.66752 × 10^−17^
hsa04068	FoxO signaling pathway	5.9291 × 10^−12^
hsa05225	Hepatocellular carcinoma	5.59347 × 10^−11^
hsa04151	PI3K-Akt signaling pathway	1.22601 × 10^−10^
hsa04010	MAPK signaling pathway	3.84059 × 10^−10^
hsa04933	AGE-RAGE signaling pathway in diabetic complications	1.33436 × 10^−9^
hsa01521	EGFR tyrosine kinase inhibitor resistance	1.31213 × 10^−8^
hsa04932	Non-alcoholic fatty liver disease	2.84255 × 10^−8^
hsa04066	HIF-1 signaling pathway	9.12132 × 10^−8^
hsa04152	AMPK signaling pathway	1.69899 × 10^−7^
hsa04915	Estrogen signaling pathway	3.69865 × 10^−7^
hsa04668	TNF signaling pathway	3.24181 × 10^−6^
hsa04150	mTOR signaling pathway	1.62932 × 10^−5^
hsa04657	IL-17 signaling pathway	4.01859 × 10^−5^
hsa04024	cAMP signaling pathway	9.32719 × 10^−5^
hsa04930	Type II diabetes mellitus	1.12074 × 10^−4^
hsa00982	Drug metabolism-cytochrome P450	4.24179 × 10^−4^
hsa03320	PPAR signaling pathway	4.78196 × 10^−4^
hsa04064	NF-kappa B signaling pathway	1.23710 × 10^−3^

We constructed a THC-target-pathway network diagram using the intersecting targets from the enriched pathways (Figure 9E). The top 15 targets ranked by descending degree value were PIK3CA, IGF1R, MAPK8, RAF1, EGFR, RPS6KB1, INSR, MAPK14, CASP3, CDK4, MET, PRKACA, TGFBR1, PPARG, and RXRA. After a literature review and comprehensive screening of the core targets, we identified six key targets (PIK3CA, IGF1R, RPS6KB1, INSR, PRKACA, and PPARG) that are significantly associated with THC’s effect in ameliorating NASH. These targets were selected for molecular docking.

### 3.5 Molecular docking and animal experimental validation

**Figure 10:**
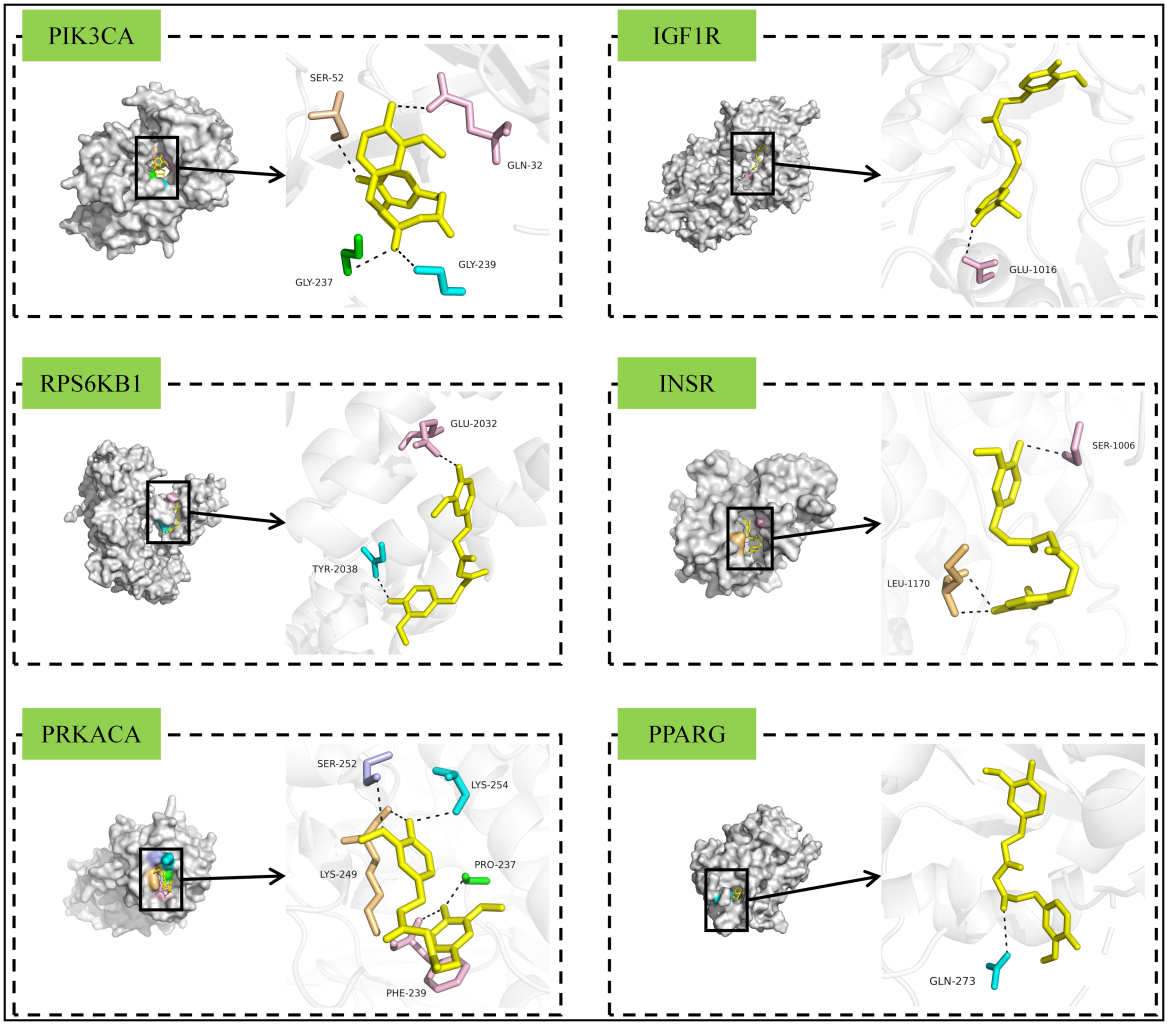
Viewable view of THC docking to each key target site.

The 3D structure of THC (CID: 124072) was downloaded from the PubChem database. Using AutoDock 4.2.6, AutoDockTools 1.5.7, AutoDock Vina 1.2.3, and PyMOL 2.6, we performed molecular docking and visualization between THC and six key targets—PIK3CA (PDB ID: 7L1C), IGF1R (1P4O), RPS6KB1 (5WBH), INSR (3BU3), PRKACA (4WB8), and PPARG (6MS7). Docking poses are visualized in Figure 10, with binding affinities summarized in Table 5. The strongest binding affinity was observed for PPARG (−6.923 kcal/mol). Gene expression analysis (Figure 9F) showed that model mice had significantly higher hepatic PPARG mRNA levels compared to controls. Low-dose THC trended toward increased PPARG expression (non-significant), whereas medium- and high-dose THC significantly decreased PPARG mRNA levels.

**Table 5:** Binding efficacy of THC to various key targets.

Component	Targets					
	PIK3CA	IGF1R	RPS6KB1	INSR	PRKACA	PPARG
THC	−6.174 kcal/mol	−6.854 kcal/mol	−6.868 kcal/mol	−6.149 kcal/mol	−5.381 kcal/mol	−6.923 kcal/mol

### 3.6 Effect of THC on mRNA of relevant targets in NASH mice

**Figure 11:**
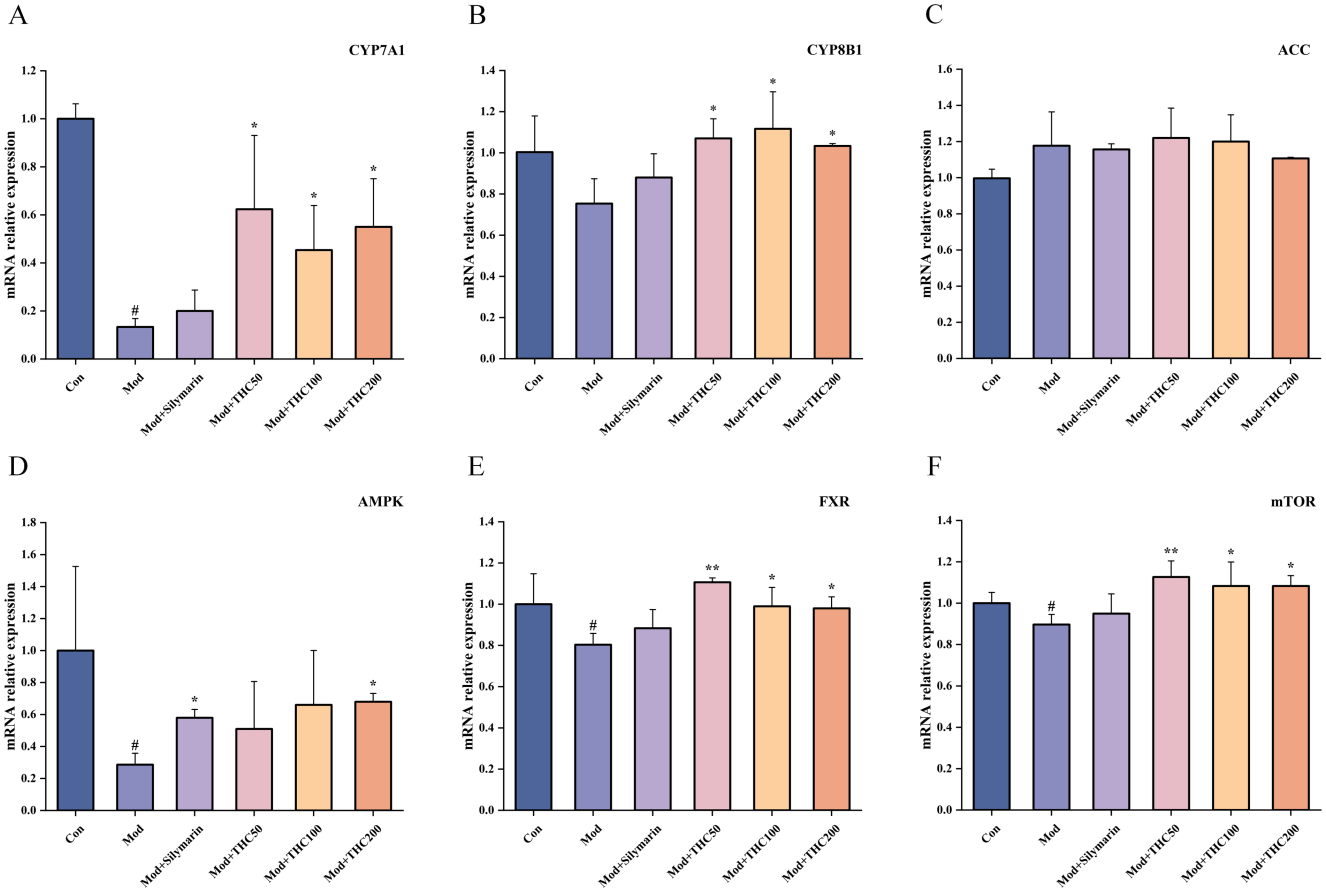
Effect of THC on mRNA expression of relevant targets in MCD mice. **(A)** Effect of THC on CYP7A1 mRNA expression in liver. **(B)** Effect of THC on CYP8B1 mRNA expression in the liver. **(C)** Effect of THC on ACC mRNA expression in liver. **(D)** Effect of THC on AMPK mRNA expression in liver. **(E)** Effect of THC on FXR mRNA expression in liver. **(F)** Effect of THC on mTOR mRNA expression in liver. Compared with Con group, ^#^*p* < 0.05; compared with Mod group, ^*^p < 0.05 and ^**^p < 0.01.

In pharmacodynamic experiments, THC exhibited cholesterol-regulating effects, as it significantly increased hepatic CYP7A1 and CYP8B1 mRNA expression (*p* < 0.05; Figures 11A,B). Additionally, THC reduced serum triglycerides (TG) and improved insulin resistance, prompting further analysis of ACC and AMPK expression (Figures 11C,D). Model mice showed increased hepatic ACC mRNA and significantly decreased AMPK mRNA compared to controls (*p* < 0.05), with high-dose THC reversing the latter by significantly elevating AMPK expression (*p* < 0.05). MCD diet feeding reduced hepatic FXR and mTOR mRNA levels (*p* < 0.05), effects reversed by THC intervention. Low-dose THC particularly enhanced FXR and mTOR expression, with significant increases observed (*p* < 0.01; Figures 11E,F).

### 3.7 Effects of THC on NASH cell models

**Figure 12:**
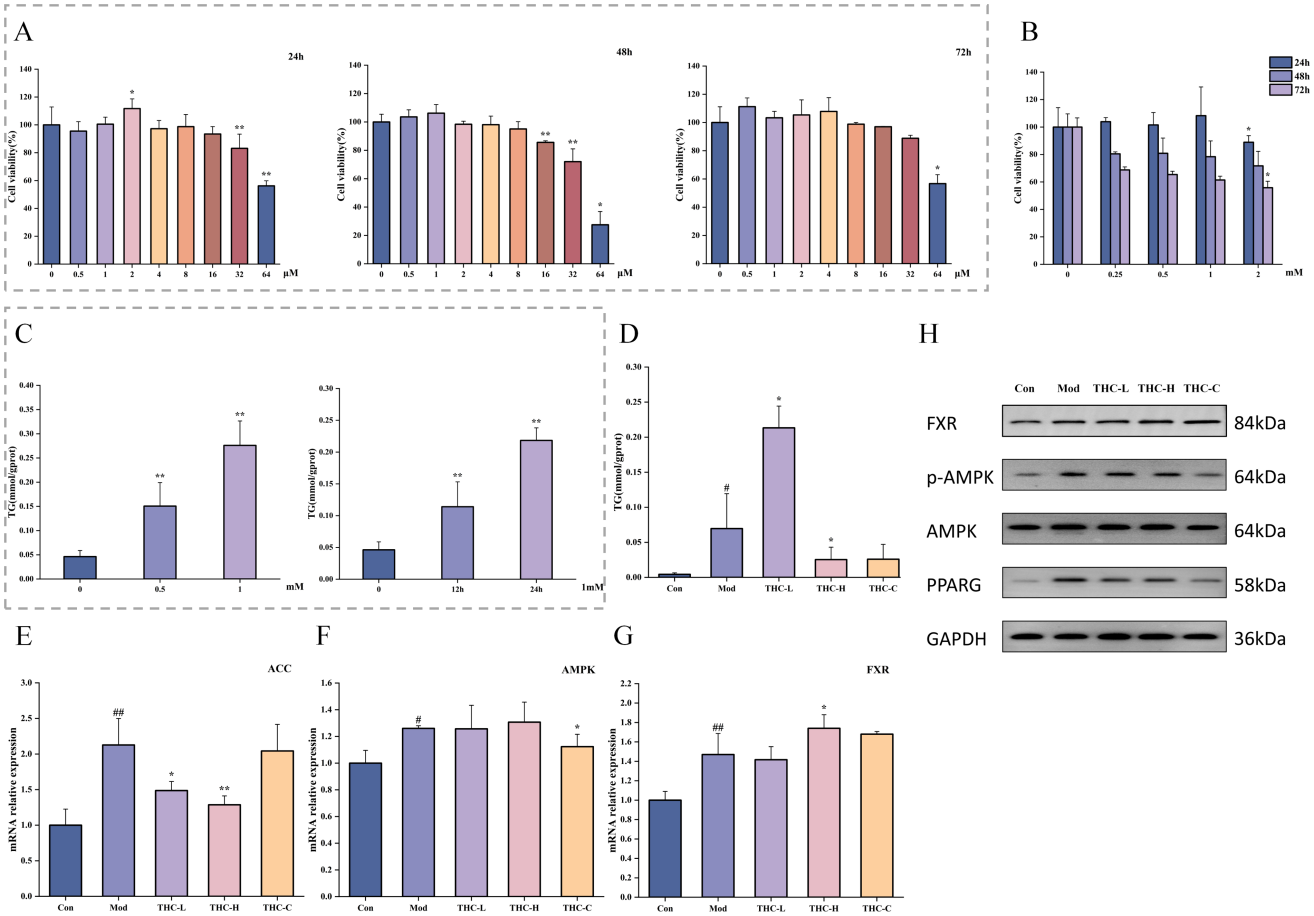
Effects of THC on NASH cell models. **(A)** Effect of different concentrations of THC on AML-12 cell viability at different time periods (*n* = 4). **(B)** Effects of different concentrations of FFAs on AML-12 cell viability at different times (*n* = 5). **(C)** Effects of different concentrations of FFAs on TG production of AML-12 cells at different time periods (*n* = 3). **(D)** Effect of THC treatment on TG production in NASH cell model induced by FFAs (*n* = 3). **(E)** Effect of THC on ACC mRNA expression in cell models (*n* = 3). **(F)** Effect of THC on AMPK mRNA expression in cell models (*n* = 3). **(G)** Effect of THC on FXR mRNA expression in cell models (*n* = 3). **(H)** Protein expression of AMPK, p-AMPK, PPARG and FXR in cells of each group. Compared with Con group, ^#^*p* < 0.05 and ^##^*p* < 0.01; compared with Mod group, ^*^p < 0.05 and ^**^p < 0.01.

Following 24 h of THC treatment, cell viability declined with increasing THC concentration. Viability at 0.5–16 μM THC was 95.54, 100.54, 111.74% (*p* < 0.05), 97.24, 98.74, and 93.44%, respectively. Concentrations exceeding 16 μM led to significant viability decreases (83.14 and 56.24% at 24 μM and 32 μM, respectively; *p* < 0.01), with identical trends observed after 48 and 72 h of treatment (Figure 12A). For FFAs, cell viability decreased with increasing concentration across treatment durations. At 24 h, 2 mM FFAs reduced viability significantly compared to 1 mM. Viability at 0.25–2 mM FFAs was 103.89, 101.57, 108.30, and 88.93%, respectively. At fixed concentrations, viability declined with treatment time: for example, 1 mM FFAs yielded 108.30% (24 h), 78.41% (48 h), and 61.46% (72 h) viability (Figure 12B). Triglyceride (TG) production increased dose-dependently after 24-h treatment with 0.5 and 1 mM FFAs (*p* < 0.01 vs. control). With 1 mM FFAs, TG production rose over time, increasing significantly after 12 and 24 h (*p* < 0.01; Figure 12C).

Based on experimental results, a 24-h incubation with 1 mM FFAs was selected to induce the NASH cell model, with 1 μM and 2 μM THC used as low and high doses, respectively. Model cells showed significantly increased triglyceride (TG) production (*p* < 0.05 vs. control), which was attenuated by high-dose THC (*p* < 0.05; Figure 12D). We next analyzed ACC, AMPK, and FXR mRNA levels. Compared to controls, model cells exhibited upregulated AMPK (*p* < 0.05) and significantly increased ACC/FXR expression (*p* < 0.01). Low-dose THC downregulated ACC mRNA (*p* < 0.05), with a more pronounced decrease at high dose (*p* < 0.01). High-dose THC also increased FXR mRNA (*p* < 0.05), an effect partially reversed by the AMPK inhibitor (Figures 12E–G).

Western blot analysis of AMPK, p-AMPK, PPARG, and FXR protein expression in AML-12 cells is shown in Figure 12H. High-dose THC increased p-AMPK protein levels and the p-AMPK/AMPK ratio compared to the model group, effects reversed by co-treatment with an AMPK inhibitor.

### 3.8 Effect of THC on the diversity of intestinal flora in NASH mice

**Figure 13:**
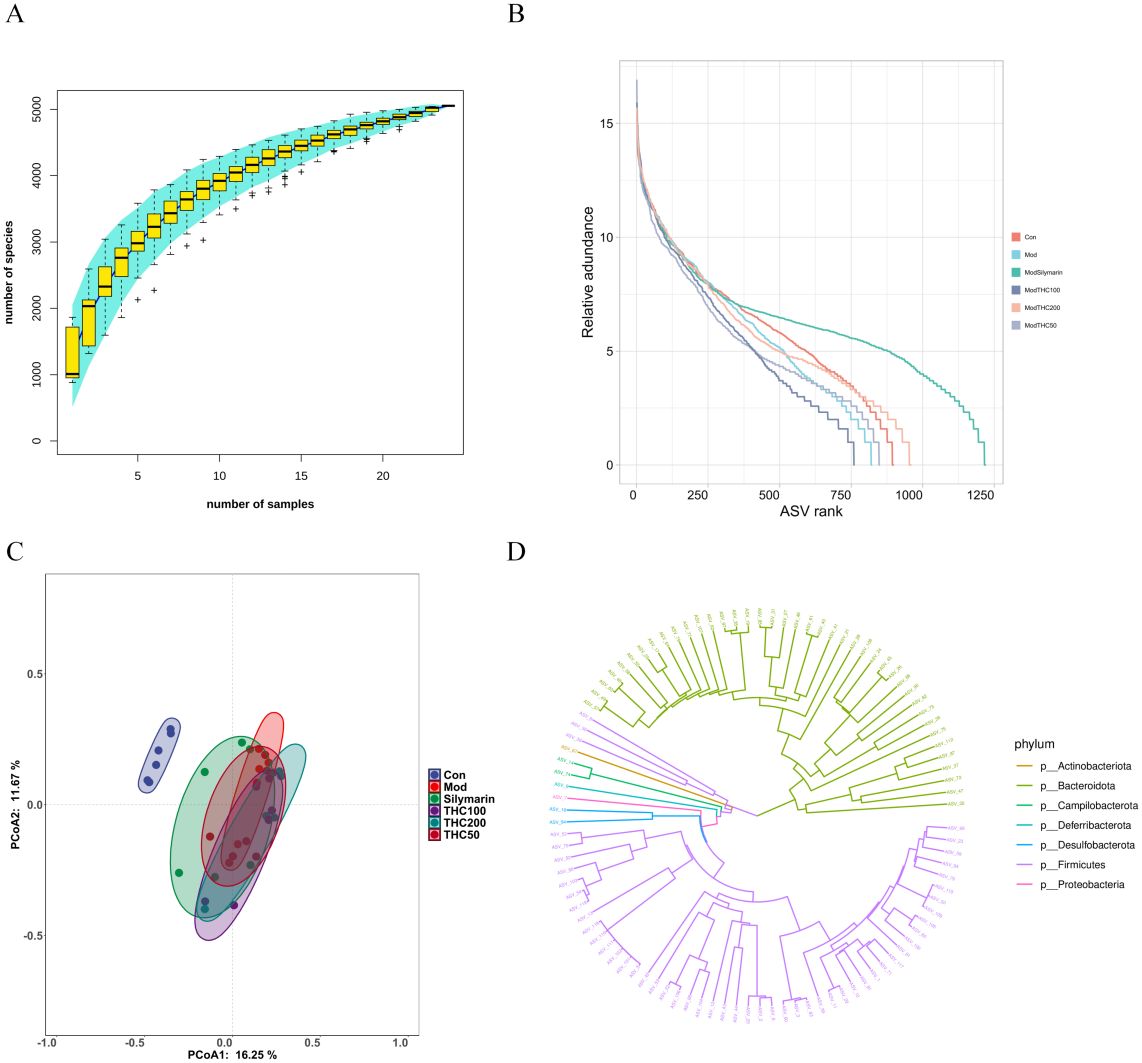
Effect of THC on the intestinal flora of NASH mice. **(A)** Species cumulative plot. **(B)** Rank abundance plot. **(C)** Beta diversity analysis. **(D)** Evolutionary tree of bacterial flora.

16S rRNA sequencing was performed on fecal samples from each mouse group. The rarefaction curve in Figure 13A rises sharply with increasing sequencing depth, then plateaus as sample size increases, indicating no new species are detected. This confirms adequate sampling and sufficient sequencing depth for subsequent analysis. Rank-abundance plots (Figure 13B) show that the high-dose THC group exhibited species abundance and evenness more similar to the control group than other experimental groups.

Alpha diversity indices (ACE, Shannon, Chao1, and Simpson) were calculated at a uniform sequencing depth (Table 6). Model mice exhibited significantly lower ACE, Shannon, Chao1, and Simpson scores (*p* < 0.01 vs. control), reflecting reduced bacterial abundance and diversity due to NASH modeling. THC treatment significantly restored Shannon and ACE indices compared to the model group. Beta diversity analysis via principal coordinate analysis (PCoA) showed PCoA1 and PCoA2 accounted for 16.25 and 11.67% of variance, respectively (Figure 13C). Intestinal flora profiles of NASH model mice clustered separately from controls, with no overlap, while the THC-treated group partially overlapped with the model group but trended toward the control cluster.

**Table 6:** Effect of THC on the alpha diversity of intestinal flora in NASH mice.

Group	Chao1	Shannon	Simpson	ACE
Con	286.96 ± 14.26	6.34 ± 0.18	0.98 ± 0.00	318.64 ± 37.41
Mod	225.10 ± 47.06^##^	5.38 ± 0.44^##^	0.93 ± 0.05^##^	243.09 ± 35.70^##^
Mod + Silymarin	272.11 ± 116.15	5.99 ± 0.69	0.96 ± 0.02	271.79 ± 115.77
Mod + THC50	249.94 ± 52.70	5.65 ± 0.26	0.96 ± 0.01	249.90 ± 52.63
Mod + THC100	242.24 ± 42.75	5.47 ± 0.38	0.94 ± 0.03	260.28 ± 39.42
Mod + THC200	238.63 ± 24.17	5.31 ± 0.59	0.94 ± 0.03	238.84 ± 24.20

Compared with Con group, ##*p* < 0.01.

### 3.9 Effect of THC on species abundance of intestinal flora in NASH mice

**Figure 14:**
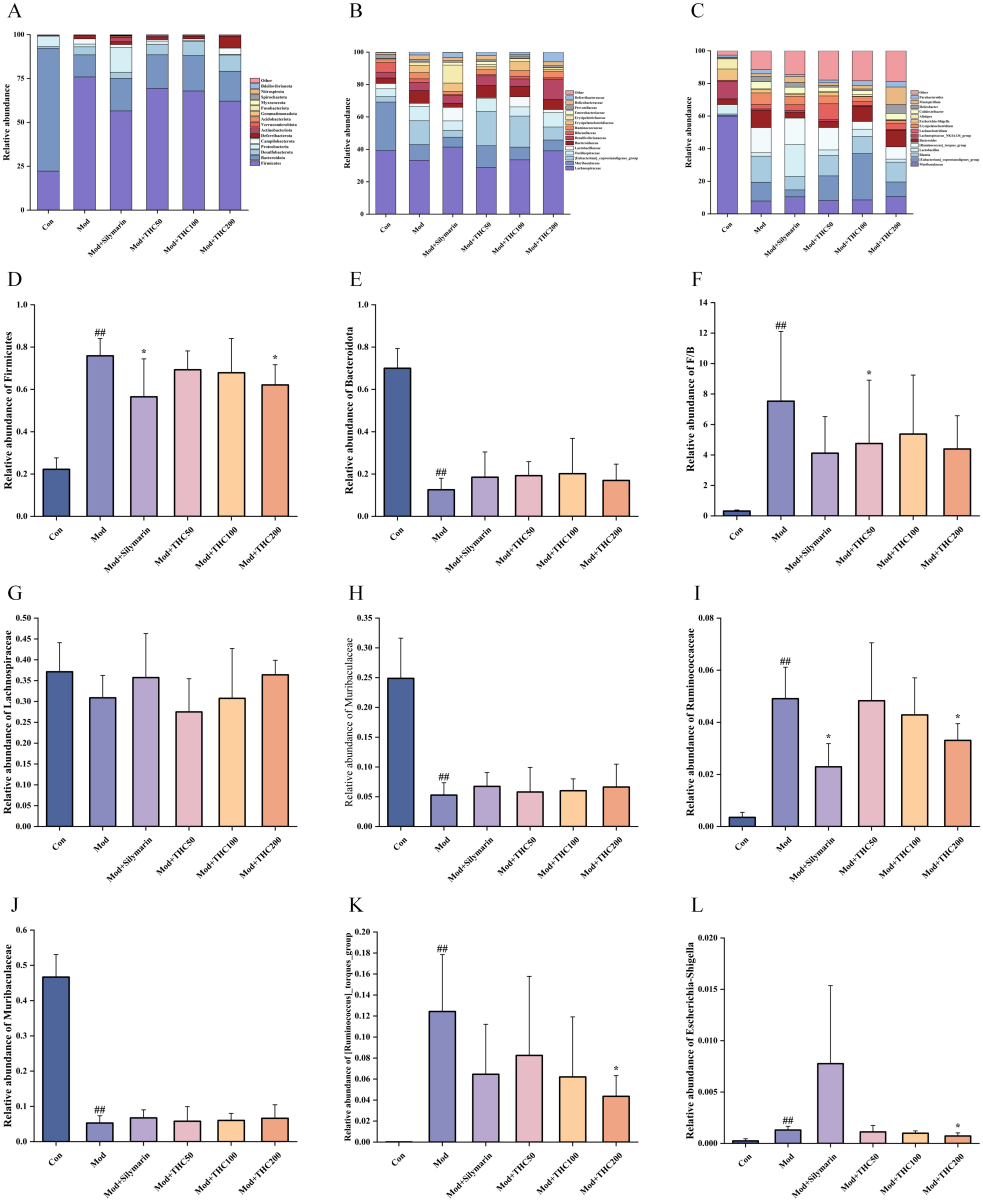
Effect of THC on intestinal flora of NASH mice at different levels. **(A)** Effect of THC on intestinal flora at the gate level. **(B)** Effect of THC on intestinal flora at the family level. **(C)** Effect of THC on intestinal flora at the genus level. **(D)** Effect of THC on the relative abundance of *Firmicutes*. **(E)** THC affects the relative abundance of *Bacteroidota*. **(F)** THC affects the ratio of *Firmicutes*/*Bacteroidota*. **(G)** THC affects the relative abundance of *Lachnospiraceae*. **(H)** THC affects the relative abundance of *Muribaculaceae*. **(I)** THC affects the relative abundance of *Ruminococcaceae*. **(J)** THC affects the relative abundance of *Muribaculaceae*. **(K)** THC affects the relative abundance of *[Ruminococcus]_torques_group*. **(L)** THC affects the relative abundance of *Escherichia-Shigella*. Compared with Con group, ##*p* < 0.01; compared with Mod group, *p < 0.05.

The phylogenetic tree (Figure 13D) showed that *Firmicutes* and *Bacteroidota* were the dominant phyla in the intestinal microbiota of all mouse groups. At the phylum level, the top 15 taxa by relative abundance were selected for the stacked bar plot (Figure 14A), revealing that gut microbiota across groups primarily consisted of *Firmicutes*, *Bacteroidota*, *Desulfobacterota*, and *Proteobacteria*. Compared with the control group, long-term high-fat diet induced significant gut microbiota structural changes in mice. The model group exhibited significantly higher *Firmicutes* relative abundance (*p* < 0.01), lower *Bacteroidota* relative abundance (*p* < 0.01), and elevated F/B ratio (*p* < 0.01). THC treatment reversed these alterations (Figures 14D–F). Specifically, low-dose THC tended to decrease *Firmicutes* and increase *Bacteroidota*, significantly reducing the F/B ratio (*p* < 0.05).

At the family level, the top 15 taxa by relative abundance were selected for the stacked bar plot (Figure 14B). Gut microbiota across groups primarily included *Lachnospiraceae*, *Muribaculaceae*, *[Eubacterium]_coprostanoligenes_group*, *Oscillospiraceae*, *Lactobacillaceae*, and other families. Compared to the control group, the high-fat diet decreased the relative abundance of beneficial bacterial families *Lachnospiraceae* and *Muribaculaceae* while significantly increasing that of the harmful *Ruminococcaceae* (*p* < 0.01). THC treatment tended to increase *Lachnospiraceae* and *Muribaculaceae* levels and decrease *Ruminococcaceae* abundance (Figures 14G–I).

At the genus level, the top 15 taxa by relative abundance were selected for stacked bar plots (Figure 14C). Gut microbiota across groups primarily included beneficial genera *Muribaculaceae, [Eubacterium]_coprostanoligenes_group*, Blautia, *Lactobacillus*, and harmful genera *[Ruminococcus]_torques_group*, *Escherichia-Shigella*, among others. Compared with the control group (Figures 14J–L), model group samples showed significantly lower relative abundance of the beneficial genus *Muribaculaceae* (*p* < 0.01) and significantly higher abundance of deleterious genera *[Ruminococcus]_torques_group* and *Escherichia-Shigella* (*p* < 0.01). THC treatment tended to increase *Muribaculaceae* abundance and significantly decreased *[Ruminococcus]_torques_group* and *Escherichia-Shigella* levels (*p* < 0.05).

### 3.10 Correlation analysis of intestinal flora with biochemical indicators and prediction of KEGG function

**Figure 15:**
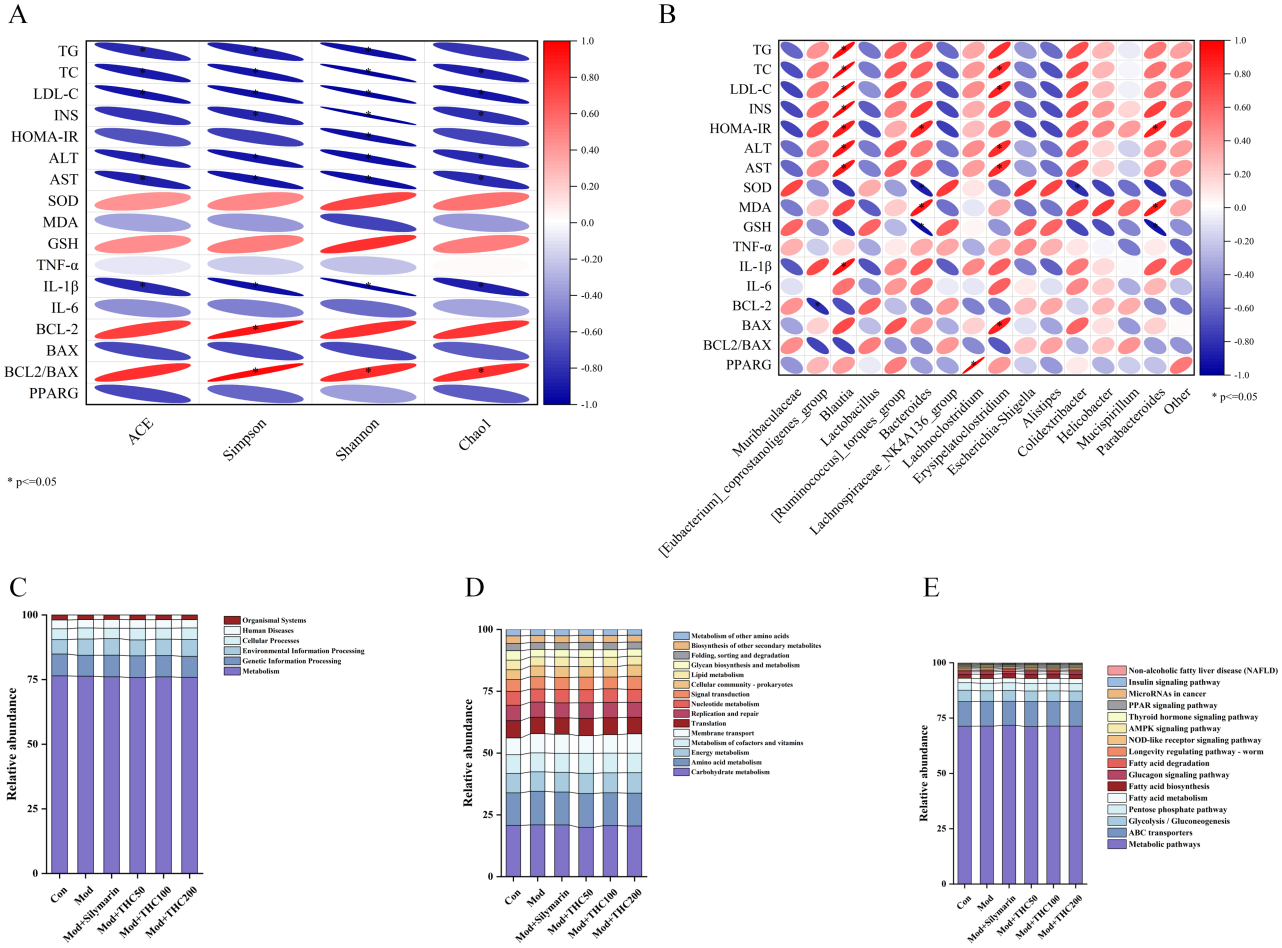
Correlation of intestinal flora with biochemical indicators and KEGG prediction. **(A)** Correlation of alpha diversity with biochemical indices. **(B)** Correlation of bacterial genera with biochemical indicators. **(C)** Level 1. **(D)** Level 2. **(E)** Level 3.

Pearson correlation analysis was performed to assess associations between intestinal microbiota alpha diversity and biochemical indices, with results visualized in Figure 15A. Alpha diversity metrics correlated negatively with serum parameters, where the Chao1 index showed significant negative associations with TG, TC, LDL-C, INS, HOMA-IR, and AST. Figure 15B displays genus-level correlations with biochemical indicators. The harmful genus *Blautia* exhibited significant positive associations with lipid levels, insulin resistance, and inflammatory markers. *Bacteroides* correlated significantly with insulin resistance indices. Beneficial genera *Muribaculaceae*, *Lactobacillus*, and *Alistipes* showed negative correlations, whereas harmful taxa like *[Ruminococcus]_torques_group* and *Colidextribacter* were positively associated with lipid, liver function, and inflammatory factors.

KEGG pathway predictions were analyzed across three hierarchical levels, with functional annotations becoming more specific at higher levels. At Level 1, gut microbiota functions across groups were primarily enriched in metabolic pathways (Figure 15C). Level 2 analysis revealed that carbohydrate metabolism, amino acid metabolism, and energy metabolism dominated the metabolic pathway category (Figure 15D). At Level 3, 369 pathways were predicted, with the most abundant including metabolic pathways, ABC transporters, and the AMPK signaling pathway (Figure 15E). Notably, the AMPK and PPAR signaling pathways identified here align with the KEGG enrichment results from the network pharmacology analysis, highlighting conserved molecular mechanisms.

## 4 Discussion

THC, an active metabolite of curcumin and a natural compound in turmeric (*Curcuma longa*) rhizomes (Lai et al., 2020), exhibits diverse pharmacological activities. Its well-characterized effects include hypolipidemic, hypoglycemic (Murugan and Pari, 2006), antioxidant (Li et al., 2019), and anti-inflammatory properties (Pan et al., 2020). Preclinical studies have also reported THC’s therapeutic benefits in metabolic and hepatic disorders, such as drug-induced liver injury (Luo et al., 2019), type 2 diabetes (Yuan et al., 2020), and alcoholic fatty liver disease (Lee et al., 2022). These findings highlight the rationale for investigating THC’s efficacy in non-alcoholic steatohepatitis (NASH).

In this study, THC effectively mitigated NASH in high-fat diet-fed C57BL/6J mice, reducing hepatic steatosis, fibrosis, dyslipidemia, oxidative stress, inflammation, and hepatocyte apoptosis. Mechanistic investigations revealed that THC modulated PPARG expression and reduced deleterious gut microbiota abundance, promoting liver repair as evidenced by decreased ALT and AST levels. These findings highlight THC as a potential therapeutic candidate for NASH.

The high-fat diet (HFD)-induced NASH model in C57BL/6 J mice represents a nutritionally dysregulated phenotype, often associated with hyperlipidemia, obesity, and metabolic syndrome components linked to insulin resistance (IR). Its pathogenesis—characterized by IR-driven metabolic and immune abnormalities from overnutrition—closely mirrors human NASH (Lieber et al., 2004). Following the methodology of Ni et al. (2020) with modifications based on prior research, we successfully replicated the NASH mouse model, validated by liver morphology and histopathological staining results.

To investigate THC’s mechanisms against NASH, we employed network pharmacology and molecular docking. Database screening identified 233 potential THC targets and 1,596 NASH-associated targets, with 61 common targets derived from their intersection. Six key targets—PIK3CA, IGF1R, RPS6KB1, INSR, PRKACA, and PPARG—were selected as critical for THC’s therapeutic effects. Phosphoinositide 3-kinases (PI3Ks) are a family of phospholipid kinases classified into types IA, IB, II, and III based on structural features. Type IA PI3K complexes comprise catalytic subunits (p110α, p110β, p110δ; encoded by PIK3CA, PIK3CB, PIK3CD) and regulatory subunits (p85α, p50α, p55α, p85β, p55γ; encoded by PIK3R1, PIK3R2, PIK3R3) (Cheng et al., 2022). PI3K functions in the *PI3K/AKT signaling pathway*, where activation alleviates hepatic inflammation and autophagy while inhibiting fibrosis markers (e.g., collagen deposition) via downstream mTOR signaling (Liu et al., 2020; Wang et al., 2019). The insulin-like growth factor 1 receptor (IGF-1R) regulates glucose metabolism, cell proliferation, and other physiological processes. Preclinical studies show that IGF-1R activation suppresses HIF-1 signaling, reducing metabolic dysfunction, hepatic steatosis, and inflammation in high-fat diet-fed mice (Zheng et al., 2021). Ribosomal protein S6 kinase beta-1 (RPS6KB1), a downstream effector of MTORC1, was investigated here. Knockdown of RPS6KB1 enhanced lipolysis rates and improved energy expenditure in mice, suggesting that low RPS6KB1 expression in model mice—observed at the mRNA level—may contribute to their reduced body mass compared with control and treatment groups (Um et al., 2004). For insulin receptor (INSR), genetic mutations impair receptor function, disrupting insulin signaling and promoting insulin resistance, a key driver of NAFLD pathogenesis (Nobakht et al., 2020). Regarding protein kinase A catalytic subunit alpha (PRKACA), it mediates SIK inactivation and CRTC2-p300–driven transcription, processes linked to hepatocellular carcinoma growth. Activation of the PRKACA/AP-1 axis further promotes cancer cell migration and invasion (Gritti et al., 2025; Liu et al., 2024). qRT-PCR showed that THC significantly downregulated PPARG mRNA expression. Previous reports indicate that PPARG modulates lipid metabolism by regulating cholesterol homeostasis and fatty acid oxidation genes, thereby correcting metabolic dysfunction (Choi et al., 2021). However, another study reported that targeted deletion of PPARG inhibited hepatic steatosis in NAFLD mice, while PPARG was highly expressed in NAFLD patients, suggesting its role in promoting steatosis (Yang et al., 2020). The mechanism may involve PPARG-mediated upregulation of fatty acid binding protein, lipoprotein lipase, and lipocalin in adipocytes, thereby enhancing lipogenesis (Kim et al., 2018). Additionally, oxidative stress promotes Nrf2 recruitment to the PPARG promoter, activating PPARG, inducing adipogenesis, and accelerating NAFLD progression (Zhong et al., 2022). In our study, THC effectively alleviated high-fat diet–induced oxidative stress, which may indirectly reduce PPARG mRNA expression. Concurrently, THC intervention attenuated hepatic steatosis and fibrosis, mimicking the effects of the PPARG antagonist GW9662, which improves lipid metabolism in NASH mice (Xiao et al., 2023). These findings suggest that THC may ameliorate NASH by downregulating PPARG expression.

Numerous studies have established a robust link between gut microbiota and metabolic diseases, with accumulating evidence showing their involvement in NASH pathogenesis (Ding et al., 2019). Dysbiosis-driven elevations in circulating lipopolysaccharides trigger endotoxemia, allowing harmful substances to translocate to the liver via the gut-liver axis and accelerate NASH progression (Tripathi et al., 2018). Additionally, microbial alterations elevate short-chain fatty acids, endogenous ethanol production, and disrupt bile acid metabolism, collectively influencing NASH development (Abdelmegeed et al., 2012).

The Farnesoid X receptor (FXR) is a key regulator of bile acid metabolism in the gut-liver axis. Preclinical studies have shown that in NAFLD models, FXR activation upregulates SREBP1c, IRS-1, and TNF-α while downregulating AMPK, exacerbating metabolic stress, dyslipidemia, and inflammatory/oxidative stress via downstream target regulation (Clifford et al., 2021). Conversely, FXR activation can ameliorate steatohepatitis and fibrosis by dynamically regulating lipid and glucose homeostasis in the NASH liver through cytokine-mediated mechanisms (Fiorucci et al., 2020). Peroxisome proliferator-activated receptor gamma (PPARG), a PPAR isoform, governs diverse metabolic pathways (Loviscach et al., 2000; Dubois et al., 2017). Preclinical evidence shows that obese mice exhibit elevated PPARG expression, with PPARG agonists worsening hepatic steatosis; conversely, genetic deletion or downregulation of PPARG protects against steatosis (Gao et al., 2016; Moran et al., 2011). In this study, THC increased hepatic FXR protein levels while decreasing PPARG expression, consistent with serum improvements in TG, TC, LDL-C, and INS in model mice—effects potentially linked to the FXR/PPARG signaling axis. Cytochrome P450 7A1 (CYP7A1), a key rate-limiting enzyme in cholesterol-to-bile acid conversion, was upregulated at the mRNA level by THC, though not fully normalized—likely due to inhibitory effects of elevated FXR mRNA on CYP7A1 transcription (Ramírez-Pérez et al., 2017).

Alpha and beta diversity analyses of mouse gut microbiota revealed that chronic high-fat diet (HFD) significantly reduced microbial abundance/diversity and altered community structure. The *Firmicutes*/*Bacteroidota* (F/B) ratio, a recognized marker of dysbiosis, was disrupted in NASH (Jasirwan et al., 2021; Lee et al., 2021). Model mice exhibited elevated *Firmicutes* and reduced *Bacteroidota* at the phylum level, driving a marked increase in F/B ratio—evidence of gut microbiota dysbiosis. THC treatment significantly lowered F/B ratio, shifting the microbiota toward a healthier state. While THC improved alpha diversity, beta diversity changes trended toward normalization, potentially contributing to NASH amelioration. Preclinical evidence links *Muribaculaceae* reduction in obese mice to increased body weight, adiposity, and plasma/hepatic lipid levels (Cao et al., 2020). Consistent with this, HFD decreased *Muribaculaceae* relative abundance, an effect reversed by THC—correlating with reduced serum TG, TC, and LDL-C. Correlation analyses showed inverse relationships between *Muribaculaceae* and dyslipidemia markers. Gram-negative bacterial overgrowth, such as *Escherichia-Shigella*, can compromise intestinal barrier integrity, promoting endotoxin translocation and chronic inflammation (Yin et al., 2013). In this study, HFD increased *Escherichia-Shigella* abundance, which positively correlated with hepatic TNF-α expression—an association mitigated by THC intervention.

To our knowledge, this study is the first to report THC hepatoprotective effects in a NASH mouse model. We integrated network pharmacology and gut microbiota analysis to investigate THC therapeutic actions against NASH, demonstrating its ability to counteract high-fat diet–induced hepatic injury. However, the specific molecular and microbial mechanisms underlying these effects remain to be fully elucidated, representing important avenues for future research.

## 5 Conclusion

**Figure 16:**
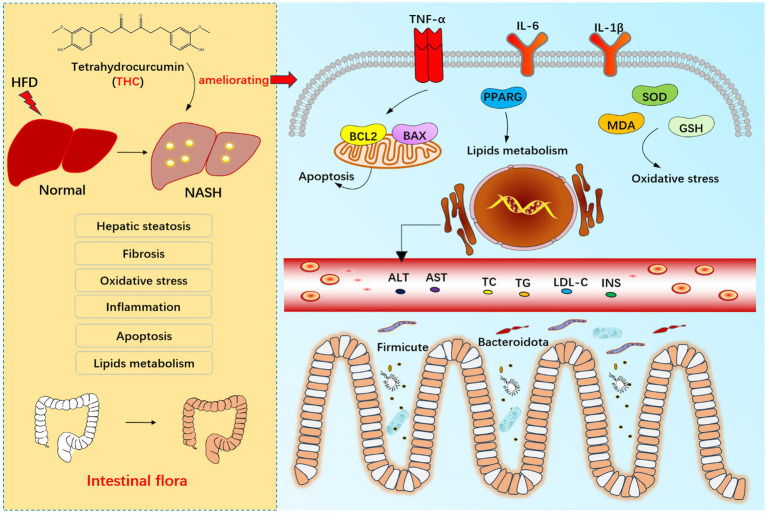
Exploring the mechanisms of THC in ameliorating nonalcoholic steatohepatitis based on network pharmacology and gut microbiota analysis *in vivo* and *in vitro*.

THC may exert anti-NASH effects through multiple pathways, including ameliorating liver injury, regulating lipid metabolism, improving insulin resistance, mitigating oxidative stress, attenuating inflammation, and enhancing anti-apoptotic capacity. The proposed mechanism involves inhibiting PPARG expression and reducing harmful gut microbiota abundance (Figure 16). This study offers a novel perspective on leveraging THC for NASH therapy.

## Data Availability

The raw data supporting the conclusions of this article will be made available by the authors, without undue reservation.
