# Correction: Exploring the mechanisms of tetrahydrocurcumin in ameliorating nonalcoholic steatohepatitis based on network pharmacology and gut microbiota analysis *in vivo* and *in vitro*

**DOI:** 10.3389/fmicb.2025.1656225

**Published:** 2025-08-07

**Authors:** Keyu Chen, Jianbo Wang, Shuang Luo, Yunyun Quan, Ping Wei, Jiali Fu, Jiali Ma, Yuying Yang, Yunten Liu, Zhichong Gao

**Affiliations:** ^1^Pharmacology of Chinese Medicine, Shaanxi University of Chinese Medicine, Xianyang, China; ^2^Translational Chinese Medicine Key Laboratory of Sichuan Province, Sichuan Institute for Translational Chinese Medicine, Sichuan Academy of Chinese Medicine Sciences, Chengdu, China; ^3^Key Laboratory of Pharmacodynamics and Material Basis of Chinese Medicine of Shaanxi Administration of Traditional Chinese Medicine, Xianyang, China; ^4^Engineering Research Center of Brain Health Industry of Chinese Medicine, Universities of Shaanxi Province, Xianyang, China; ^5^College of Food and Biological Engineering, Chengdu University, Chengdu, China; ^6^School of Pharmacy, Southwest Medical University, Luzhou, China

**Keywords:** nonalcoholic steatohepatitis, tetrahydrocurcumin, network pharmacology, 16S rRNA, intestinal flora imbalance

In the published article, there were several errors in the author affiliations given. The affiliations of each author in the original article have been corrected as follows:

° The affiliation “Translational Chinese Medicine Key Laboratory of Sichuan Province, Sichuan Institute for Translational Chinese Medicine, Sichuan Academy of Chinese Medicine Sciences, Chengdu, China” has been moved from the fourth position in the affiliation list to the second position. The remaining affiliations have been reordered accordingly.

The updated affiliation list as follows: Keyu Chen 1,3,4, Jianbo Wang 1,2*, Shuang Luo 5, Yunyun Quan 2, Ping Wei 2, Jiali Fu 5, Jiali Ma 5, Yuying Yang 6, Yunten Liu 5 and Zhichong Gao 1,3,4

1 Pharmacology of Chinese Medicine, Shaanxi University of Chinese Medicine, Xianyang, China

2 Translational Chinese Medicine Key Laboratory of Sichuan Province, Sichuan Institute for Translational Chinese Medicine, Sichuan Academy of Chinese Medicine Sciences, Chengdu, China

3 Key Laboratory of Pharmacodynamics and Material Basis of Chinese Medicine of Shaanxi Administration of Traditional Chinese Medicine, Xianyang, China

4 Engineering Research Center of Brain Health Industry of Chinese Medicine, Universities of Shaanxi Province, Xianyang, China

5 College of Food and Biological Engineering, Chengdu University, Chengdu, China

6 School of Pharmacy, Southwest Medical University, Luzhou, China

The original version of this article has been updated.

